# Comparative efficacy of nine exercise methods on the prognosis in chronic kidney disease patients with hemodialysis: a systematic review and network meta-analysis

**DOI:** 10.1186/s40001-023-01270-9

**Published:** 2023-10-05

**Authors:** Ning Ren, Huiting Yang, Zelin Cai, Ruye Wang, Zeng Wang, Ying Zhao, Chenyun Miao, Yun Chen, Yang Zhang, Xingyu Zhu, Hongyu Chen, Qin Zhang

**Affiliations:** 1 Hangzhou TCM Hospital of Zhejiang Chinese Medical University (Hangzhou Hospital of Traditional Chinese Medicine), Hangzhou, 310007 Zhejiang China; 2 School of Public Health, Zhejiang Chinese Medical University, Hangzhou, 310053 Zhejiang China; 3 School of Life Sciences, Zhejiang Chinese Medical University, Hangzhou, 310053 Zhejiang China; 4 Department of Medical TCM Gynaecology, Hangzhou TCM Hospital of Zhejiang Chinese Medical University (Hangzhou Hospital of Traditional Chinese Medicine), Hangzhou, 310007 Zhejiang China; 5 Department of Nephrology, Wenzhou Hospital of Integrated Traditional Chinese and Western Medicine, Wenzhou, 325000 China; 6 Department of Nephrology, Hangzhou TCM Hospital of Zhejiang Chinese Medical University (Hangzhou Hospital of Traditional Chinese Medicine), Hangzhou, 310007 Zhejiang China

**Keywords:** Hemodialysis, Efficacy, Exercise, Network meta-analysis

## Abstract

**Background:**

Several kinds of physical activities have been applied to improve the prognosis of patients with hemodialysis (HD). However, the comparative efficacy of physical activities on the outcomes in HD patients is still unknown. This study explored the effectiveness and safety of all exercise types in HD patients.

**Methods:**

We searched randomized clinical trials from the PubMed, EMBASE, and Cochrane Library databases. Physical exercises interventions included resistance exercise (RE), aerobic exercise (AE), electrical muscle stimulation (EMS), range of motion (ROM), resistance exercise + aerobic exercise (RE + AE), stretching exercise (STE), respiratory muscle training (RMT), peripheral muscle training (PMT), walking exercise (WE), or usual care/sham exercise (UC/SE). Primary outcomes were six-minute walk test (6-mwt) and quality of life (QOL). Secondary outcomes were Kt/V, VO_2max_, hemoglobin (Hb), C-reactive protein (CRP), interleukin-6 (IL-6), and systolic and diastolic blood pressure (sbp and dbp). Frequentist network meta-analysis with multivariate random effects models provided mean with 95% confidence intervals (95%CI).

**Results:**

A total of 58 eligible studies were included. AE, RMT, and RE + AE significantly improved 6-mwt compared with UC/SE. SE was the worst intervention and reduced QOL much more than the UC/SE and other exercise types. AE and RE + AE were associated with higher VO_2max_, while ROM and RE + AE induced higher Hb levels. All physical activities did not elevate blood pressure, CRP and IL-6. Only ROM decreased sbp/dbp. CRP is significantly lower in RE.

**Conclusion:**

Physical activities play a crucial role in the different outcomes of HD patients. They can be applied to specific area for their specific efficacy.

**Supplementary Information:**

The online version contains supplementary material available at 10.1186/s40001-023-01270-9.

## Introduction

End-stage renal disease (ESRD) is the final stage of chronic kidney disease (CKD) patients. The incidence is becoming more prevalent, which worsens the prognosis [1]. For patients with ESRD, renal replacement therapy (RRT) is the only treatment option. In 2030, more than 5 million people worldwide will likely receive RRT [2]. RRT consists of renal transplant, hemodialysis (HD), and peritoneal dialysis. HD is the most used approach globally, accounting for almost two-thirds of all dialysis, with 22% of ESRD patients receiving a kidney transplant and 9.7% receiving peritoneal dialysis [3].

Although HD extends life expectancy, people with HD frequently experience several complications, including cardiovascular disease, renal hypertension, and decreased physical activity levels. These issues constantly worsen their symptoms and reduce the quality of their lives [4–6]. In contrast to healthy people, HD patients' physical quality gradually degrades [7]. According to a prior study, a lack of activity before and after dialysis will decrease the patient's exercise level [8]. Reduced levels of activity will raise the mortality risk [9]. In addition, some researchers have indicated that HD patients with lower activity levels are likely to have a greater risk of death [10].

Some studies concluded that intradialytic workouts enhance the prognosis of HD patients by reducing inflammation, increasing aerobic capacity, and enhancing the quality of life (QOL) [11, 12]. Yet, there are several kinds of intradialytic movements. Not all intradialytic activities have the effect described above [13, 14]. Despite prior meta-analyses assessing the impact of intradialytic exercises on HD patients, no study has compared the efficacy of various exercise procedures on the prognosis in HD patients. Investigating the influence of different exercise strategies on particular outcomes is significant. Therefore, we conducted a systematic review and network analysis to compare various exercise techniques on HD patients' prognoses.

## Methods

This study has been registered in the International Prospective Register of Systematic Reviews (CRD42023324600). We performed the network meta-analysis under the Preferred Reporting Items for Systematic Reviews and Meta-Analyses (PRISMA) guide.

### Search strategy

Two investigators independently reviewed PubMed, EMBASE, Cochrane Library, and Google Scholar from inception to Dec. 21, 2022. We searched for articles using medical topic headings (MeSH) and accessible terms without restrictions on countries, regions, races, or languages. Additional file 1: Methods S1, S2, and S3 provided search terms and tactics information. We also mentioned annual meetings and abstracts.

### Eligibility criteria

Patients who regularly underwent HD were eligible. They were treated with usual care/sham exercise (UC/SE) or physical exercise during HD sessions, including resistance exercise (RE), aerobic exercise (AE), electrical muscle stimulation (EMS), range of motion (ROM), aerobic plus resistance training (AE + RE), stretching exercise (STE), respiratory muscle training (RMT), peripheral muscle training (PMT), and walking exercise(WE). The main results included a six-minute walk distance (6-mwt) and QOL. Secondary outcomes were hemoglobin (Hb), urea clearance index (Kt/V), VO_2max_, C-reactive protein (CRP), interleukin-6 (IL-6), and systolic/diastolic blood pressure (sbp/dbp). To prevent bias, we included randomized clinical trials (RCT).

### Data extraction

Two investigators independently extracted data from included studies, and any inconsistencies were resolved by consensus with a third investigator. The following characteristic information was recorded: (1) study characteristics: first author, publication time, study design, follow-up time, training time and intervention type; (2) population information: sample size and age range; and (3) outcomes: 6-mwt, QOL, CRP, VO_2max_, systolic and diastolic blood pressure, Hb, Kt/V, IL-6. We also checked each clinical trial's supplemental papers to make sure no details were missed.

### Quality assessment

Two authors, respectively, evaluated the bias risk of included studies. using the Cochrane evaluation handbook and used a weighted Cohen’s kappa coefficient (κ) to measure agreement. The bias risk is divided into low, unclear, and high. It had randomization sequence generation (selection bias), allocation concealment (selection bias), blinding of participants and personnel (performance bias), blinding of outcome assessment (detection bias), incomplete outcome data (attrition bias), selective reporting (reporting bias), and other biases. Differences were resolved by consensus.

### Statistical synthesis and analysis

RevMan 5.3 and StataSE 17.0 were used for the study's statistical analyses (StataCorp LP, College Station, TX, USA). This study employed the frequentist method of the random effect model to conduct network meta-analyses (NMAs). Primary outcomes included 6-mwt and QOL. The secondary results were Kt/V, bdp, sbp, CRP, IL-6, Hb, and VO_2max_. Continuous variables were displayed using mean differences and 95% confidence intervals. For all comparisons, forest plots represented the summary treatment effects. Interventions can be ranked by calculating the surface under the cumulative ranking curve (SUCRA). SUCRA displays a percentage and establishes the likelihood that a workout strategy is the most efficient. The greater surface area under the curve indicates a higher possibility that a specific workout style will be the most effective intervention. We calculated statistical inconsistency using global, node-splitting, and loop inconsistencies. For global inconsistency, *p* < 0.05 was regarded to show statistically significant heterogeneity. Using the node-splitting method, *p* < 0.05 revealed a statistically significant discrepancy between direct and indirect evidence. The inconsistency factor (IF) evaluates the bias extent and inconsistency in the loop-specific technique. When the 95% CI of the IF included 0, it indicated that estimates of intervention effects derived from direct and indirect evidence are consistent.

## Result

### Eligible studies

**Fig. 1:**
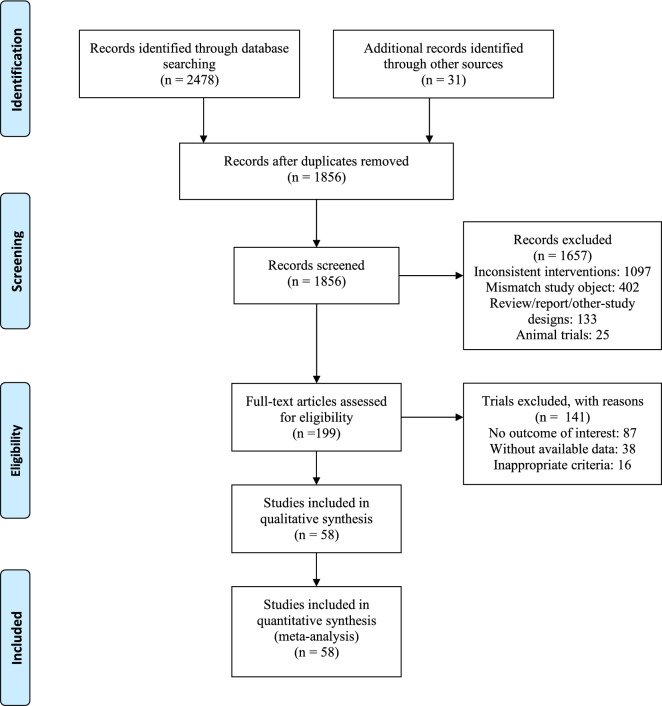
Flowchart of the study. The study followed the Preferred Reporting Items for Systematic Reviews and Meta-Analyses (PRISMA) guidelines

Figure 1 depicts the screening procedure for studies. We searched 2478 related articles in the Cochrane Library, Embase, and PubMed databases. Following the removal of 622 duplicates, 1856 articles were further evaluated. Based on examining their titles and abstracts, 1657 of these papers were eliminated as being unrelated. According to the complete text, the remaining 199 articles were further screened, and 137 were removed for failing to match the criteria. The withdrawn publications included 87 with no relevant outcome, 38 without available data, and 12 with improper standards. As a result, the meta-analysis utilized 58 eligible studies.

### Study characteristics

Additional file 1: Table S1 lists the features of the 58 qualifying studies. Nine exercise interventions used were RE, AE, EMS, ROM, RE + AE, STE, RMT, PMT, and WE. 2731 volunteers were recruited. According to the baseline data, most interventions occurred during the first 2 h of HD and the mean research duration were 4 months.

### Quality of the included studies

According to the Cochrane Collaboration tool, all studies displayed a low risk of reporting bias. No paper blinded participants or investigators. Many studies are susceptible to performance bias since blind measures were difficult to conduct. The included studies' overall grade was considered moderate (Additional file 1: Fig. S1). Additional file 1: Table S3 shows that there was high inter-rater agreement for risk of bias assessments (κ between 0.740 and 1.00 across domains).

## Network meta‐analysis

### Primary outcomes

#### 6-mwt

**Fig. 2:**
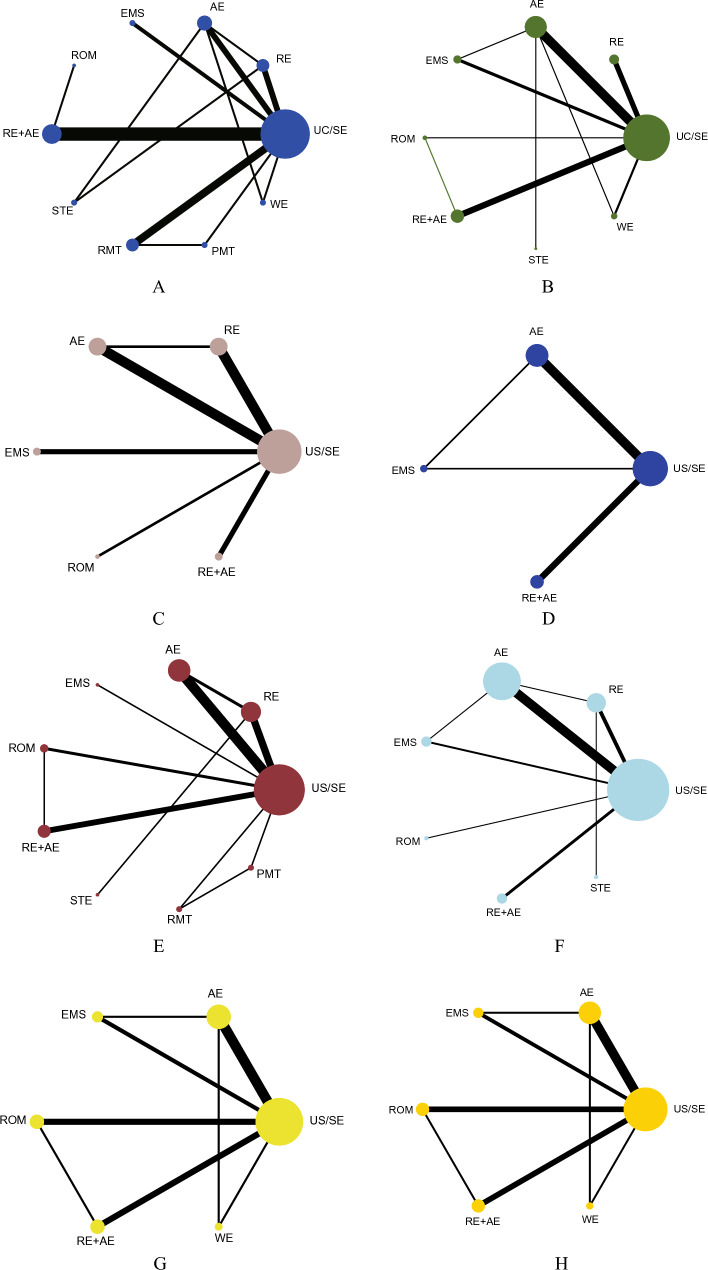
Network of physical exercises. **A** Six-minute walk test, **B** quality of life, **C** C-reactive protein, **D** VO_2max_, **E** hemoglobin, **F** Kt/V, **G** systolic blood pressure, **H** diastolic blood pressure comparing different modalities of exercise

**Fig. 3:**
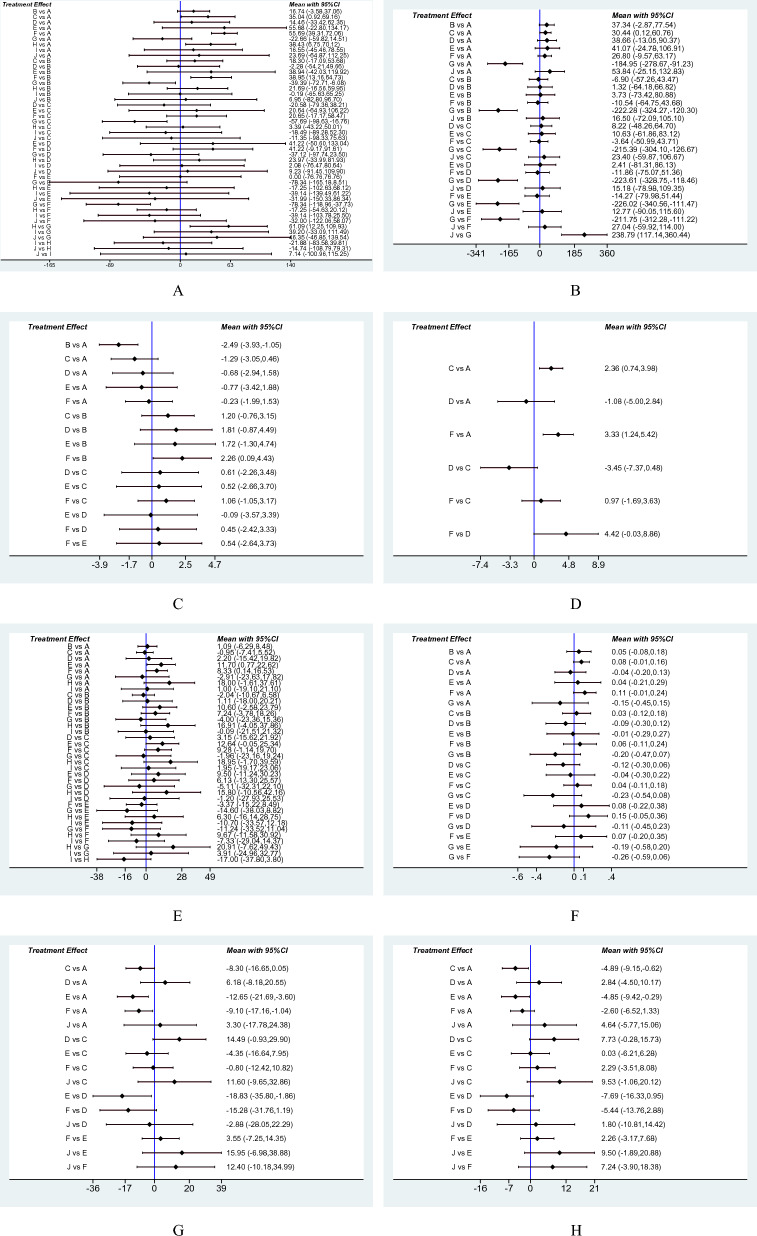
Forest plots of network meta-analysis. **A** Six-minute walk test, **B** quality of life, **C** C-reactive protein, **D** VO_2max_, **E** hemoglobin, **F** Kt/V, **G** systolic blood pressure, **H** diastolic blood pressure

**Fig. 4:**
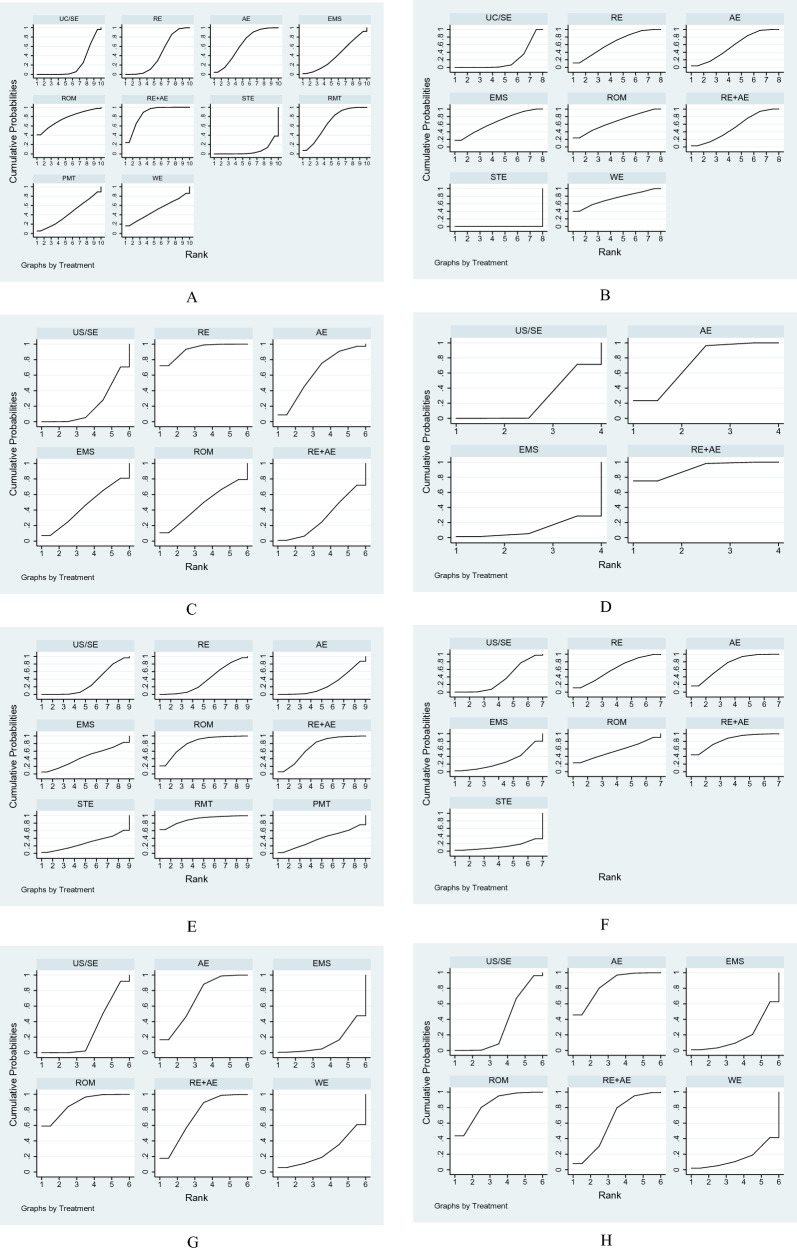
The surface under the cumulative ranking curve (SUCRA) for physical exercise interventions. **A** Six-minute walk test, **B** quality of life, **C** C-reactive protein, **D** VO_2max_, **E** hemoglobin, **F** Kt/V, **G** systolic blood pressure, **H** diastolic blood pressure

23 RCTs with 915 individuals reported changes in the 6-mwt from the baseline (Fig. 2A). We identified UC/SE intervention as a control group. Compared to the control group, the AE (35.04, 0.92 to 69.16, *p* = 0.044), RE + AE (55.69, 39.31 to 72.06, *p* < 0.001), and RMT (38.43, 6.75 to 70.16, *p* = 0.017) forms resulted in a significant 6-mwt improvement. Also, they statistically increased 6-mwt much more than the STE intervention (Fig. 3A). However, there was no distinction between these three training types' effects on the 6-mwt. RE + AE (86.2%) was ranked as the best sport intervention to increase 6-mwt for HD patients by SUCRA (Fig. 4A).

#### QOL

26 studies with 1495 participants looked into QOL (Fig. 2B). We found AE was linked to a greater QOL when compared to the control group (30.44, 0.12 to 60.76, *p* = 0.049). In addition, STE decreased QOL compared to the control group (− 184.95, − 278.67 to − 91.01, *p* = 0.001) and other exercises, including RE, AE, EMS, ROM, and RE + AE (Fig. 3B). According to the SUCRA ranking, the poorest motion in elevating QOL was STE (0.0%) (Fig. 4B).

### Secondary outcomes

Studies assessing secondary outcomes ranged in number from 4 to 20 (Fig. 2C–H**,** Additional file 1: Fig. S3). We discovered that none of the activities affected Kt/V or IL-6. Compared to the control group, CRP is significantly lower in RE (− 2.49, − 3.93 to − 1.05, *p* = 0.001). RE + AE had a greater CRP than RE (2.26, 0.09 to 4.43). The most effective method for lowering CRP in HD patients was RE (92.9%). Compared with the control group, greater VO_2max_ was caused by RE + AE and AE interventions (2.36, 0.74 to 3.98; 3.33, 1.24 to 5.42). They also ranked the first two groups regarding VO_2max_ (91.1% and 73.2%, respectively). Hb levels were raised by ROM and RE + AE (11.70, 0.77 to 22.62, p = 0.036; 8.33, 0.14 to 16.53, *p* = 0.046). When comparing the effects of the exercises on blood pressure to the control group, ROM and RE + AE resulted in lower sbp (− 12.65, − 21.69 to − 3.60, *p* = 0.051; − 9.10, − 17.16 to − 1.04, p = 0.006), while ROM and AE led to lower dbp (− 4.89, − 9.15 to − 0.62, *p* = 0.025; − 4.85, − 9.42 to − 0.29, *p* = 0.037). Also, they ranked the leading interventions. Figures 3C–H, 4C−H and Additional file 1: Fig. S4, S5 included illustrations of each forest plot and each SUCRA figure.

### Heterogeneity and inconsistency assessment

No statistically significant discrepancy was found in the results of the global inconsistency test (*p* = 0.914). The outcomes of the node-splitting method and loop-specific approach are shown in Additional file 1: Table S2 and Fig. S2, respectively. The results indicated no discrepancy between direct and indirect comparisons.

### Small-study effect analysis

**Fig. 5:**
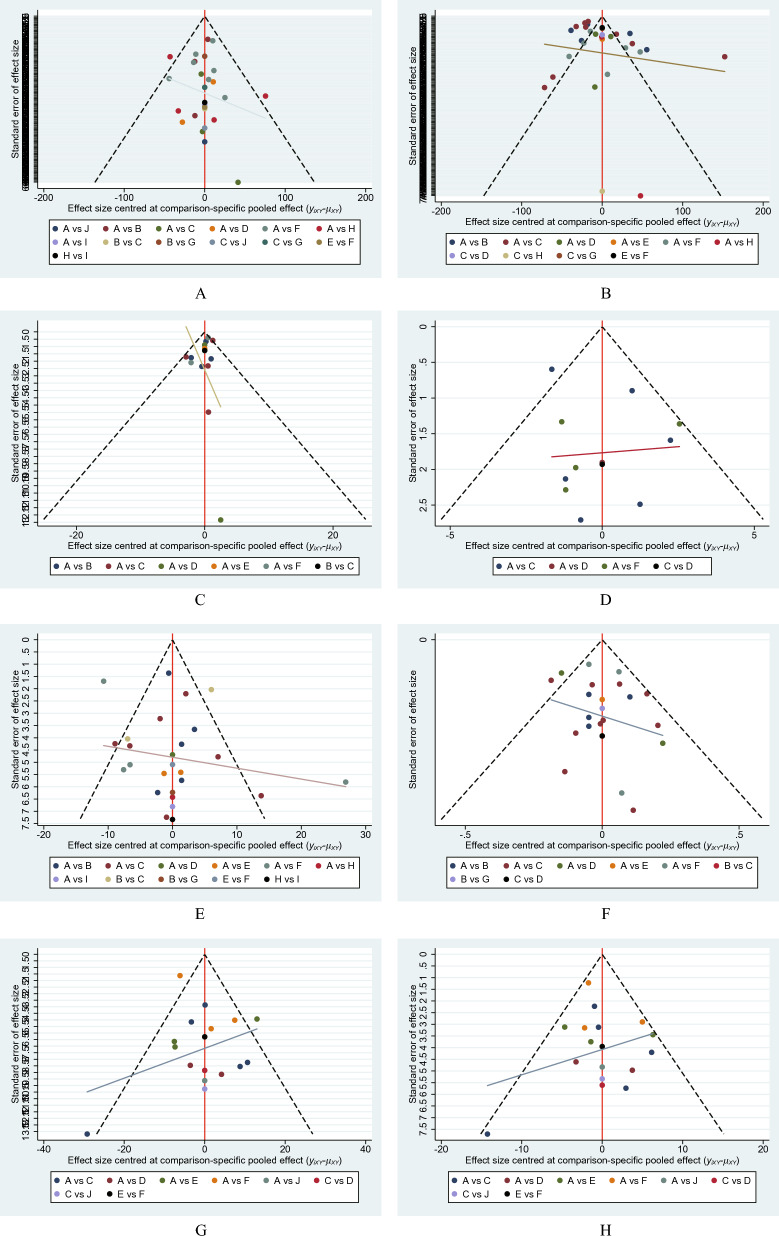
Funnel plots of the study treatments. **A** Six-minute walk test, **B** quality of life, **C** C-reactive protein, **D** VO_2max_, **E** hemoglobin, **F** Kt/V, **G** systolic blood pressure, (H) diastolic blood pressure

According to the comparison-adjusted funnel plots' findings, there might not be small-study effects for effectiveness (Fig. 5, Additional file 1: Fig S6).

## Discussion

### Principal findings

This network meta-analysis examined the effect of nine physical activities on the health outcomes of HD patients. These included the following exercises: RE, AE, EMS, ROM, RE + AE, STE, RMT, PMT, and WE. Compared to UC/SE alone, all activity interventions did not affect Kt/V or IL-6. Significant 6-mwt elevations are caused by AE, RE + AE, and RMT, with RE + AE having the most impact. AE is effective in raising QOL. Yet, STE significantly decreased QOL more than UC/SE alone. While ROM and RE + AE were linked to increased Hb, AE and RE + AE performed at higher VO_2max_. RE performed best in decreasing CRP. Only ROM simultaneously decreased systolic and diastolic pressure among nine different physical activities.

### Comparisons with other studies

Previous reviews and meta-analyses have evaluated the effects of IDE on HD patients. However, these studies did not wholly screen all available evidence. A lot of outcomes were not investigated in previous analyses. This study contained 58 RCTs to evaluate 9 exercise types. To this day, this network meta-analysis is the most extensive study investigating the influence of physical activity on HD patients.

Compared to ESRD patients without HD, HD patients have limited activity capacity. Inadequate dialysis and immobility impair their physical strength somewhat [15]. Lower exercise capacity is associated with higher mortality risk and worse prognosis [15]. Some guidelines recommend moderate-intensity exercise to improve HD patients’ exercise capacity [16]. This study considered 6-mwt and QOL as essential indicators for evaluating cardiopulmonary function and exercise ability [17–19]. VO_2max_ refers to the oxygen content patients can absorb and assess maximum exercise intensity [20]. In this study, AE, RE + AE, and RMT were demonstrated with the efficacy of elevating 6-mwt. Among these three activities, AE benefits the QOL. AE and RE + AE can increase VO_2max_. The effectiveness of AE and RE + AE in 6-mwt and VO_2max_ is consistent with previous studies [21]. Biochemical and molecular physiology analyses proved that aerobic training and combining resistance activity improve exercise capacity. Endurance training increases the number of muscle mitochondria, enhances oxidative phosphorylation, and stimulates mitochondrial biogenesis by activating the peroxisome proliferator-activated receptor g coactivator 1a signaling pathway in response to an increase in intracellular Ca^2+^ and reactive oxygen species. Increased cyclic adenosine monophosphate (AMP) and p38 mitogen-activated protein kinase result from enhanced adrenergic stimulation and adenosine triphosphate (ATP) breakdown [22]. Some clinical studies suggested HD patients with aerobic training during the first 2 h of the dialysis sessions. AE is primarily supervised stationary cycling with a moderate-to-high intensity based on VO_2max_ assessment.

Chronic inflammation is also a complication for ESRD patients. CRP and IL-6 are standard parameters used to estimate the inflammation condition [23]. An abnormal state will induce vascular calcification, cardiovascular disease [24, 25], and even accelerate aging [26]. For CRP, We demonstrated that all nine types of physical exercises did not stimulate CRP. Consistent with this outcome, three studies found a decrease after RE. Moraes et al. discovered reductions in CRP after RE, while Dong et al. found the same results after high-intensity RE [5, 27]. They presented similar results as the published meta-analysis. When RE was carried out with a high level of intensity, the effects on CRP were more apparent. A previous study also discovered a reduced CRP in medium-intensity AE intervention. However, we found no difference between AE and UC/SE. This inconsistency indicated that there is probably an association between CRP reduction and exercise intensity. A published study consistently reported this view, they supposed better results of CRP in patients who perform AE with medium-intensity training or RE with high-intensity procedures. Therefore, adjusting training intensity is vital in reducing CRP in HD patients [12]. IL-6 has proven to be a better predictor of cardiovascular mortality and general mortality in these patients [28, 29], which is associated with more inflammatory causes. Though only 4 included RCT contained available data of IL-6, and only 3 exercises were covered, the small-study effect analysis ensured the reliability of our data. In our study, AE, RE and STE would not increase or reduce IL-6. In addition, there was no statistically significant difference in the effect on IL-6 when comparing between these three exercises. This hinted that exercise did not increase the risk of death due to the inflammatory response. But there was no additional benefit in terms of reducing the inflammatory response and attenuating inflammation-induced cardiovascular deaths at the same time. It seems that we could conservatively affirm that exercise will not aggravate the effects of inflammatory state or even cardiovascular risk in dialysis patients based on the results of CRP and IL-6. Moreover, since it is difficult to measure IL-6 in clinical practice [30], other reliable and diverse inflammatory markers are needed to connect their predictive role for patient prognosis.

Hypertension is a prevalent complication for HD patients. The incidence is almost 90% worldwide [31]. Hypertension in HD patients is frequently hard to treat. 22% of those patients can still not benefit from antihypertensive drugs. Hypertension is a high-risk factor for cardiovascular disease and all-cause mortality in dialysis patients [32]. As we know, blood pressure inevitably rises during physical activity. This study examined the safety of movements on systolic/diastolic pressure and found no significant blood pressure elevation compared to usual care/sham exercise in nine activities. A meta-analysis supported this little influence on hypertension. However, single studies have found varying and inconclusive effects of several exercise training types on HD patients' blood pressure levels [33]. Some investigators found a sbp reduction different from 4 to 10 mmHg in AE and a dbp decrease from 3 to 6 mmHg in AE + RE. On the contrary, in this study, we discovered sbp and dbp reduction in AE + RE and AE, respectively. Only ROM reduces sbp/dbp at the same time. Most studies did not report the measurement methods of blood pressure. Differences in blood pressure measurement approaches may explain these divergences [21]. Although the blood pressure effect is controversial, we can confirm that physical activity does not increase blood pressure and is safe for HD patients with hypertension.

HD is vital in prolonging ESRD patients’ survival. Kt/V is a sensitive indicator applied to measure dialysis adequacy. Most countries recommend a target dose of 1.2–1.4 [34]. Although exercise was reported to improve dialysis efficacy by increasing blood flow, diffusing the toxins and urea into circulation, and enlarging surface area [35], this study did not identify the overall effect of physical activity on Kt/V based on the mechanism. Consistent with us, other meta-analyses [36, 37] did not show a statistically significant impact on the change in Kt/V (MWD 0.2, 95% CI –0.12 to 0.28). Nada et al. [37] reported that AE had no positive effect on Kt/V. Duration and physical status are critical to Kt/V improvement with IDE. According to the mechanism above, sp Kt/V could be improved in a single intervention part. Most trials observed Kt/V at the beginning and end-up timepoint. Rare studies traced Kt/V throughout the experiments, which may account for the controversies between different studies. Besides, Kt/V is related to residual renal function. Although patients accept the HD method to clear urea and other elements, many still have residual renal function. The difference in residual renal function may also be a reason for inconsistent Kt/V.

## Conclusions

This network meta-analysis compared the effectiveness of nine physical activities on prognosis outcomes in HD patients. Exercises improving 6-mwt include AE, AE + RE, and RMT. Among these three activities, AE and AE + RE can help HD patients to achieve higher VO_2max_. Unlike AE, STE is unsuitable for HD patients because of its poor effect on QOL. ROM and AE + RE can be applied to increase Hb and reduce systolic pressure. Patients with higher diastolic pressure can choose AE and ROM. ROM is the best intervention for patients with higher systolic and diastolic pressure. All physical exercises are not associated with Kt/V and do not increase CRP and blood pressure.

## Limitations

Some limitations of this review should not be neglected. The effect of physical activity on CRP still needs further investigation. In the future, we will explore whether exercise intensity performs lower CRP. Participants enrolled in this network meta-analysis are HD patients. The findings of this study are not suitable for patients with non-HD.

### Supplementary Information

**Additional file 1:** **Method S1.** Search strategy for PubMed. **Method S2.** Search strategy for Cochrane. **Method S3.** Search strategy for Embase. **Table S1.** Characteristics of studies and subjects included in the review. **Table S2.** Node-splitting approach for inconsistency assessment of all comparisons. **Table S3.** Risk of bias of randomized controlled trials. **Figure S1.** Quality assessment of the included studies. **Figure S2.** Loop-specific approach for inconsistency assessment of all comparisons. **Figure S3.** Network of physical exercises for IL-6. **Figure S4.** Forest plots of network meta-analysis for IL-6. **Figure S5.** The surface under the cumulative ranking curve (SUCRA) for physical exercise interventions for IL-6. **Figure S6.** Funnel plots of the study treatments for IL-6.

## Data Availability

The datasets generated during and analyzed during the current study are available from the corresponding author on reasonable request.
