# Supplementary material for: Comparative efficacy of nine exercise methods on the prognosis in chronic kidney disease patients with hemodialysis: a systematic review and network meta-analysis
Source: Eur J Med Res. 2023 Oct 5;28:401. doi: 10.1186/s40001-023-01270-9 (PMC10552225; doi:10.1186/s40001-023-01270-9)
Supplement: Supplementary file 1 — Additional file 1: Method S1. Search strategy for PubMed. Method S2. Search strategy for Cochrane. Method S3. Search strategy for Embase. Table S1. Characteristics of studies and subjects included in the review. Table S2. Node-splitting approach for inconsistency assessment of all comparisons. Table S3. Risk of bias of randomized controlled trials. Figure S1. Quality assessment of the included studies. Figure S2. Loop-specific approach for inconsistency assessment of all comparisons. Figure S3. Network of physical exercises for IL-6. Figure S4. Forest plots of network meta-analysis for IL-6. Figure S5. The surface under the cumulative ranking curve (SUCRA) for physical exercise interventions for IL-6. Figure S6. Funnel plots of the study treatments for IL-6. [file 40001_2023_1270_MOESM1_ESM.docx]

Additional file

**Method**

**Method S1** - Search strategy for PubMed

**Method S2** - Search strategy for Cochrane

**Method S3** - Search strategy for Embase

**Table**

**Table S1** – Characteristics of studies and subjects included in the review

**Table S2** – Node-splitting approach for inconsistency assessment of all comparisons

**Table S3** – Risk of bias of randomized controlled trials

**Figure S1 –** Quality assessment of the included studies

**Figure S2 –** Loop-specific approach for inconsistency assessment of all comparisons

**Figure S3 –** Network of physical exercises for IL-6

**Figure S4 –** Forest plots of network meta-analysis for IL-6

**Figure S5 –** The surface under the cumulative ranking curve (SUCRA) for physical exercise interventions for IL-6

**Figure S6 –** Funnel plots of the study treatments for IL-6

**Method S1** - Search strategy for PubMed

#1 ("Renal Dialysis"[Mesh]) OR (Dialysis) OR (Dialyses, Renal) OR (Renal Dialyses) OR (Dialysis, Renal) OR (Hemodialysis) OR (Hemodialyses) OR (haemodialysis) OR (hemodialyses) OR (hemodiafiltrat*) OR (Dialysis, Extracorporeal) OR (Dialyses, Extracorporeal) OR (Extracorporeal Dialyses) OR (Extracorporeal Dialysis) OR (“Renal Replacement Therapy"[Mesh]) OR (renal replacement therap*) OR (kidney replacement therap*) OR (kidney support) OR (renal support)

#2 ("Exercise"[Mesh] OR "Exercise Therapy"[Mesh] OR "Resistance Training"[Mesh] OR "Muscle Stretching Exercises"[Mesh]) OR (Exercises) OR (Physical Activity) OR (Activities, Physical) OR (Activity, Physical) OR (Physical Activities) OR (Exercise, Physical) OR (Exercises, Physical) OR (Physical Exercise) OR (Physical Exercises) OR (Exercise, Aerobic) OR (Aerobic Exercise) OR (Aerobic Exercises) OR (Exercises, Aerobic) OR (Exercise Training) OR (Exercise Trainings) OR (Training, Exercise) OR (Trainings, Exercise) OR (Therapy, Exercise) OR (Exercise Therapies) OR (Therapies, Exercise) OR (Rehabilitation Exercise) OR (Exercise, Rehabilitation) OR (Exercises, Rehabilitation) OR (Rehabilitation Exercises)

#3 ("Randomized Controlled Trial" [Publication Type] OR "Controlled Clinical Trial" [Publication Type] OR "Clinical Trials as Topic"[Mesh:NoExp] OR randomized[Title/Abstract] OR placebo [Title/Abstract] OR randomly[Title/Abstract] OR trial[Title/Abstract]) NOT ("Animals"[Mesh] NOT "Humans"[Mesh])

#1 AND #2 AND #3

**Method S2** - Search strategy for Cochrane

#1 MeSH descriptor: [Renal Dialysis] explode all trees OR MeSH descriptor: [Dialysis] explode all trees OR (hemodialysis):ti,ab,kw OR (haemodialysis):ti,ab,kw OR (hematodialysis):ti,ab,kw OR (renal dialysis):ti,ab,kw OR (renal support):ti,ab,kw OR (kidney support):ti,ab,kw OR (kidney replacement therap):ti,ab,kw OR (renal replacement therap):ti,ab,kw OR (kidney dialysis):ti,ab,kw OR (dialysis):ti,ab,kw

#2 (exercise*):ti,ab,kw OR (physical activit*):ti,ab,kw OR (aerobic exercise*):ti,ab,kw OR (resistance exercise*):ti,ab,kw OR (stretching exercise):ti,ab,kw OR (motion):ti,ab,kw OR (movement):ti,ab,kw OR (aerobic training):ti,ab,kw OR (oxygen sport):ti,ab,kw OR (oxygen-enriched exercise):ti,ab,kw OR (resistance training):ti,ab,kw OR (stretching training):ti,ab,kw OR (resistance movement):ti,ab,kw

OR MeSH descriptor: [Exercise] explode all trees OR MeSH descriptor: [Resistance Training] explode all trees OR MeSH descriptor: [Exercise Therapy] explode all trees

#1 AND #2

**Method S3** - Search strategy for Embase

#1 'dialysis'/exp OR ‘hemodialysis’/exp OR haemodialysis OR hemodialysis:ti,ab,kw OR hematodialysis:ti,ab,kw OR 'renal replacement therapy':ti,ab,kw OR dialysis:ti,ab,kw OR 'renal dialysis':ti,ab,kw OR 'extracorporeal dialysis’:ti,ab,kw

#2 'exercise'/exp OR 'aerobic exercise'/exp OR 'resistance training'/exp OR exercise* OR 'resistance training':ti,ab,kw OR 'aerobic exercise*':ti,ab,kw OR 'aerobic training':ti,ab,kw OR 'physical fitness':ti,ab,kw OR 'physical training':ti,ab,kw OR 'exercise therapy':ti,ab,kw OR 'combined training':ti,ab,kw OR 'aerobic plus strength training':ti,ab,kw OR 'aerobic plus resistance exercise*':ti,ab,kw OR 'aerobic plus resistance training':ti,ab,kw OR 'concurrent training':ti,ab,kw OR 'concurrent exercise*':ti,ab,kw OR electrostimulation:ti,ab,kw OR 'electro stimulation':ti,ab,kw OR 'inspiratory muscle training':ti,ab,kw OR 'motion exercise*':ti,ab,kw

#3 'crossover procedure':de OR 'double-blind procedure':de OR 'randomized controlled trial':de OR 'single-blind procedure':de OR (random* OR factorial* OR crossover* OR cross NEXT/1 over* OR placebo* OR doubl* NEAR/1 blind* OR singl* NEAR/1 blind* OR assign* OR allocat* OR volunteer*):de,ab,ti

#1 AND #2 AND #3

**Table S1** – Characteristics of studies and subjects included in the review

| Author year | Frequency, times/week | No. of Patients | Median age, years | Duration, months | Group | Group | Regimen | Quality of life measurement |
| --- | --- | --- | --- | --- | --- | --- | --- | --- |
| Depaul 2002[1] | 3 | 20 | 55±16 | 50.4±57.6 | Intervention arm | AE+RE | 2 min warm-up, cycling for 20-min and isotonic quadriceps and hamstrings RT at a level of perceived exertion at approximately 50rpm | SF-36 |
|  |  | 18 | 54±14 | 55.2±54 | Control arm | ROM | Rang of motion of the lower extremities and free upper extremity |  |
| Parsons 2004[2] | 3 | 6 | 60±17 | 35±25 | Intervention arm | AE | Three 15 min bouts of IDC at 40–50% of their maximum work capacity | SF-36 |
|  |  | 7 | 49±25 | 49±26 | Control arm | UC/SE | UC |  |
| Vilsteren 2005[3] | 2-3 | 53 | 52±15 | 39±49 | Intervention arm | AE+RE | 5–10 min warm-up, 20 min pre-dialysis strength training, and 5–10 min cool-down. 20–30 min IDC with 12–16 RPE | SF-36 |
|  |  | 43 | 58±16 | 47±53 | Control arm | UC/SE | UC |  |
| Cheema 2007[4] | 3 | 24 | 60.0±15.3 | 39.6 | Intervention arm | RE | Two sets of 10 exercises at 15 to 17/20 on the Borg Scale using free weights | SF-36 |
|  |  | 25 | 65.0±12.9 | 19.2 | Control arm | UC/SE | UC |  |
| Matsumoto 2007[5] | 3 | 17 | 60.8±9.5 | 148.8±81.6 | Intervention arm | AE | 2 to 5 min warm-up and stretching exercises followed by 20 min continuous cycling. | SF-36 |
|  |  | 32 | 57.2±8.3 | 152.4±90 | Control arm | UC/SE | UC |  |
| Ouzouni 2009[6] | 3 | 18 | 47.4±15.7 | 92.4±84 | Intervention arm | AE | 5 min warm-up, 20–60 min IDC with Borg’s scale 13–14; 5 min cool-down; and 30 min strengthening and flexibility exercises | SF-36 |
|  |  | 13 | 50.5±11.7 | 103.2±72 | Control arm | UC/SE | UC |  |
| Orti 2009[7] | / | 17 | 53.5±18 | 37.3±34.9 | Intervention arm | RE | Three sets of 4 exercises at an intensity of 12–15 using weights and elastic bands on every session | SF-36 |
|  |  | 8 | 60.1±16.9 | 53.7±42.0 | Intervention arm | AE | Cycling at a constant low work- load, so that the intensity was equivalent to an RPE of 11 |  |
| Afshar 2010[8] | 3 | 7 | 51±16.4 | 24.86±18.69 | Intervention arm | RE | 5 min warm up, a 10–30-min RE training, and a 5-min cool down | / |
|  |  | 7 | 50.7±21.06 | 25.71±7.61 | Intervention arm | AE | 5 min warm up, a 10–30-min AE training, and a 5-min cool down |  |
|  |  | 7 | 53±19.4 | 24.86±15.44 | Control arm | UC/SE | UC |  |
| Reboredo 2010[9] | 3 | 11 | 49.6±10.6 | 41.9±42.4 | Intervention arm | AE | 15 minutes warm up, 35 minutes conditioning according to the tolerance of each patient, and 3 minutes cool down | / |
|  |  | 11 | 43.5±12.8 | 60.1±54.4 | Control arm | UC/SE | UC |  |
| Dobsak 2011[10] | 3 | 11 | 58.2±7.2 | 49.2±25.2 | Intervention arm | AE | 5 min warm-up, 20–40 min IDC at the level of 60% of the individual Wpeak, 5 min cool-down | SF-36 |
|  |  | 11 | 64.5±8.1 | 46.8±21.6 | Intervention arm | EMS | EMS stimulation (10 Hz) of leg extensors wasapplied for 60 min |  |
|  |  | 10 | 60.1±8.2 | 49.2±27.6 | Control arm | UC/SE | UC |  |
| Makhlough 2012[11] | 3 | 25 | 53.30±14.27 | 25.50±10.70 | Intervention arm | AE | 15 minutes AE of motion joints was started and continued based on patients capacity | / |
|  |  | 23 | 56.16±10.77 | 23.47±13.59 | Control arm | UC/SE | UC |  |
| Song 2012[12] | 3 | 20 | 52.1±12.4 | 38.9±26.1 | Intervention arm | RE | PRT for 30 minutes with gradually increasd exercise intensity with elastic bands and sandbags | SF-36 |
|  |  | 20 | 54.6±10.1 | 45.9±56.2 | Control arm | UC/SE | UC |  |
| Delima 2013[13] | 3 | 11 | 49.6±9.1 | 64.8±48 | Intervention arm | RE | 3 series of 15 repetitions in lower limb utilizing 40% of load of a 1RM | / |
|  |  | 10 | 43.1±13.3 | 76.8±52.8 | Intervention arm | AE | Cycling for 20 min at an intensity of modified Borg scale between 2-3 |  |
|  |  | 11 | 43.5±11.1 | 78±50.4 | Control arm | UC/SE | UC |  |
| Mohseni 2013[14] | 3 | 23 | 53±14 | 26±11 | Intervention arm | AE | 15 min AE of ROM | / |
|  |  | 24 | 56±11 | 24±14 | Control arm | UC/SE | UC |  |
| Pellizzaro 2012[15] | / | 14 | 48.9±10.1 | 54±80.92 | Intervention arm | PMT | Trained the knee extensor muscles | / |
|  |  | 11 | 43±13.8 | 60±80 | Intervention arm | RMT | Three sets of 15 inspirations at the equipment mouthpiece and rested for 60 seconds according to 50% of PImax |  |
|  |  | 14 | 51.9±11.6 | 54±48.89 | Control arm | UC/SE | UC |  |
| Wu 2014[16] | 3 | 32 | 45±8.15 | 55.5±37.3 | Intervention arm | AE | 5 min warm-up and 10–15 min of IDC, Borg score of 12–16 | SF-36 |
|  |  | 33 | 44±6.67 | 39.8±29.7 | Control arm | UC/SE | SE |  |
| Liao 2016[17] | 3 | 20 | 62±8 | 71±46 | Intervention arm | AE | 5 min warm-up, 20 min of cycling at a desired workload, and a 5 min cool down | / |
|  |  | 20 | 62±9 | 83±71 | Control arm | UC/SE | UC |  |
| Roxo 2016[18] | 3 | 20 | 46.40±15.43 | 68.80±46.90 | Intervention arm | EMS | 30 min femoral quadriceps muscles electrical stimulation | / |
|  |  | 20 | 54.65±19.93 | 46.15±41.40 | Control arm | UC/SE | UC |  |
| Frih 2017[19] | 4 | 21 | 64.2±3.4 | 72.7±12.7 | Intervention arm | AE+RE | 10 min warm-up, 20 min dynamic closed and open-chain strengthening exercises, 20 min ergometer cycling and treadmill walking, 10 min cool down | SF-36 |
|  |  | 20 | 65.2±3.1 | 73.6±13.4 | Control arm | UC/SE | UC |  |
| Manfredini 2016[20] | / | 104 | 63±13 | / | Intervention arm | WE | home-based walking exercise | KDQOL-SF |
|  |  | 123 | 64±14 | / | Control arm | UC/SE | UC |  |
| Campos 2018[21] | / | 29 | 48.86±12.97 | 29.97±21.29 | Intervention arm | RMT | 12 sessions lasting 30 min each and resistance of 15 cmH2O, followed by 12 sessions lasted 40 min each and resistance was set at 20 cmH2O | / |
|  |  | 12 | 52.67±14.61 | 23.58±19.70 | Control arm | UC/SE | UC |  |
| Mcgregor 2018[22] | 3 | 16 | 52.1±7.85 | / | Intervention arm | AE | One hour cycling to an RPE of 12–14 | / |
|  |  | 17 | 51.5±9.15 | / | Intervention arm | EMS | 30 min low-frequency EMS stimulation |  |
|  |  | 18 | 54.3±8.25 | / | Control arm | UC/SE | UC |  |
| Suzuki 2018[23] | 3 | 13 | 66.2±12.8 | 28.1±24.2 | Intervention arm | EMS | Each duty cycle included a 5-s stimulation period with a 2-s pause for a period of 20 min using a monophasic, exponential climbing pulse | SF-8 |
|  |  | 13 | 65.1±8.1 | 30.4±23.6 | Control arm | UC/SE | UC |  |
| Dong 2019[24] | 3 | 21 | 59.0±25.19 | 69±41.48 | Intervention arm | RE | 10 × 10 lower limb raising REx and 10×10 up limb bouncy ball REx with less than 15 in Borg scale | / |
|  |  | 20 | 62.5±14.44 | 53±40 | Control arm | UC/SE | UC |  |
| Fernandes 2019[25] | 3 | 20 | 44.25±11.30 | 79.8±56.4 | Intervention arm | AE | 10 min warm up, 30 min ergometer activity cycle between 50 and 70% of its maximum | / |
|  |  | 19 | 42.63±11.1 | 85.92±45.36 | Control arm | UC/SE | UC |  |
| Hatef 2020[26] | 3 | 28 | 52.07±11.3 | 43.32±32.28 | Intervention arm | AE+RE | 5 min warm-up, 20 min walk, and 5 min cool-down | / |
|  |  | 27 | 53.96±10.01 | 54.24±56.52 | Control arm | UC/SE | UC |  |
| Huang 2020[27] | 3 | 16 | 43.81±10.25 | 26±29.75 | Intervention arm | AE+RE | 5 min warm up, 30 min cycling at a RPE of 12-14, and 5 min cool-down | SF-36 |
|  |  | 16 | 37.63±10.31 | 43±89 | Control arm | UC/SE | UC |  |
| Valle 2020[28] | 3 | 12 | 49.3±12.4 | 81.6±136.8 | Intervention arm | RE | Two sets of 10 repetitions of each exercise in the first week; three sets of 10 repetitions in the 2–12 week. | / |
|  |  | 12 | 60.4±10.6 | 46.8±120 | Control arm | STE | Performed on the dialysis chair and directed to the following muscles: hamstring, hip adductors, hip abductors, tibialis anterior, gastrocnemius and soleus |  |
| Yeh 2020[29] | 3 | 30 | 57.87±13.21 | 63.37±71.98 | Intervention arm | AE+RE | 5 min warm up, 20 min cyclying from motor level 2 to 4 to 2 at the perceived exertion scale of 12–14, 5 min cool dowm | / |
|  |  | 32 | 53.91±12.60 | 78.28±63.95 | Control arm | UC/SE | UC |  |
| Zhang 2020[30] | 2 | 43 | 60.0 ±11.1 | 39.0±48.89 | Intervention arm | RE | RPE 8–10 for 5 min warm-up and cool down, 30–40 min of exercise at a RPE of 10–13 on the Borg scale | SF-36 |
|  |  | 44 | 62.0±10.37 | 30.5±44.07 | Control arm | UC/SE | UC |  |
| Fathi 2021[31] | 3 | 15 | 53.86±2.50 | / | Intervention arm | AE | 30 to 45 min IDE with a maximum intensity of 50–70% of the maximum heart rate stored on the mini-bike | SF-36 |
|  |  | 15 | 54.53±2.16 | / | Control arm | UC/SE | UC |  |
| Greenwood 2021[32] | 3 | 135 | 60.5±15.0 |  | Intervention arm | AE | Cycling from short bouts of 8–10 minutes to bouts of 21 minutes and bouts of 40 minutes or more | SF-36 |
|  |  | 145 | 59.8±14.1 |  | Control arm | UC/SE | UC |  |
| Lin 2021[33] | 3 | 32 | 62.0±9.5 | 80.4±68.4 | Intervention arm | AE | 5 min warm up，20 min endurance at 12–14 based on the Borg’s Perceived Exertion Rating Scale, and 5 min cool down | SF-36 |
|  |  | 32 | 62.1±12.3 | 74.4±61.2 | Control arm | UC/SE | UC |  |
| March 2022[34] | 3 | 46 | 53±15 | 26±29 | Intervention arm | RE | 30 minutes continuous cycling at Borg RPE 12-14 | / |
|  |  | 46 | 61±14 | 25±32 | Control arm | UC/SE | UC |  |
| Rochmawati 2021[35] | 2 | 24 | 46.83±7.839 | 50.04±2.64 | Intervention arm | ROM | 15 min ROM with each movement condUC/SEted 20 rounds per minute | SF-36 |
|  |  | 24 | 48.58±11 .225 | 51.6±3.9 | Control arm | UC/SE | UC |  |
| Assawasaksakul 2021[36] | / | 6 | 52.5±12.9 | 105±92.81 | Intervention arm | AE+RE | Cycling at 80–100 RPMs with RPE or Borg’s scale up to 12 and then increasing each week until 30 min | WHOQOL-BREF |
|  |  | 6 | 53.7±17.2 | 66.5±51.70 | Control arm | UC/SE | UC |  |
| Castaneda 2004[37] | 3 | 14 | 65±9 | / | Intervention arm | AE | 5 min warm up, 35 min RE at 80% of 1 repetition maximum, and 5 min cool down | / |
|  |  | 12 | 64±12 | / | Control arm | STE | Stretching exercise |  |
| Jamshidpour2019[38] | 2 | 15 | 64.93±7.79 | 37.68± | Intervention arm | RE+AE | 3–5 min warm-up, 20 to 45 min IDC with the 11–15 Borg scale, and then lower extremity resistance exercise | SF-36 |
|  |  | 13 | 58.46±11.85 | 40.56±33.36 | Control arm | UC/SE | UC |  |
| Painter 2002[39] | 3 | 12 | 43.56±10.5 | 60.4±80.0 | Intervention arm | AE | Cycling for 30 min at the intensity of PRE level between 12- 14 | SF-36 |
|  |  | 12 | 50.16±13.8 | 67.8±54.4 | Control arm | UC/SE | UC |  |
| Soliman 2015[40] | 3 | 18 | / | / | Intervention arm | ROM | 15 min ROM according to patients tolerance with each action of 20 RPM | / |
|  |  | 12 | / | / | Control arm | UC/SE | UC |  |
| Abreu 2017[41] | 3 | 25 | 45.7±15.2 | 71.2±45.5 | Intervention arm | RE | Three sets of 10 repetitions with four movements using ankle-cuffs and elastic bands in lower limbs. 60% of 1-repetition maximum test | SF-36 |
|  |  | 19 | 42.5±13.5 | 70.1±49.9 | Control arm | UC/SE | UC |  |
| Hristea 2016[42] | 3 | 7 | 68.5±13.97 | 9.16±4.00 | Intervention arm | AE | 30 min cycling at intensity of 3-moderate on the Rating Perceived Exertion | SF-36 |
|  |  | 9 | 70.8±15.18 | 8.18±3.95 | Control arm | UC/SE | UC |  |
| Koufaki 2002[43] | 3 | 18 | 57.8±14.3 | 41.4±45.2 | Intervention arm | AE | Two separate bouts of continuous cycling of 20 min each on the cycle ergometer, or one of 30–35 min duration | / |
|  |  | 15 | 51 ± 18.9 | 53.4±52.5 | Control arm | UC/SE | UC |  |
| Kouidi 2009[44] | 3 | 24 | 46.3 ± 11.2 | 73.2±55.2 | Intervention arm | AE+RE | 5-min warm-up, a 30–60 min active cycling session, a 20-min strengthening program, and a 5-min cool-down period. | / |
|  |  | 20 | 45.8 ± 10.9 | 75.6±58.8 | Control arm | UC/SE | UC |  |
| Marchesan 2014[45] | 3 | 11 | / | / | Intervention arm | AE+RE | 20 min AE at moderate model of Borg Scale | SF-36 |
|  |  | 11 | / | / | Control arm | UC/SE | UC |  |
| Marchesan 2016[46] | 3 | 8 | 63.2±3.5 | / | Intervention arm | AE+RE | AE in a stationary bicycle, followed by RE, and gradually increasing over time | SF-36 |
|  |  | 7 | 64.7±5.2 | / | Control arm | UC/SE | UC |  |
| Schardong 2017[47] | 3 | 11 | 59.00±20 | 47.10±44.61 | Intervention arm | EMS | Starting EMS at 20 min and increasing 2 min per week | / |
|  |  | 10 | 64.50±7.59 | 56.18±61.60 | Control arm | UC/SE | UC |  |
| Poorsaadet 2018[48] | 3 | 27 | 47 | / | Intervention arm | AE | Five min exercises followed with 12–14 severity according to Borg Measure for 30 min | / |
|  |  | 11 |  | / | Control arm | UC/SE | UC |  |
| Rosa 2018[49] | 3 | 28 | 54.49 ± 11.97 | 18±15 | Intervention arm | RE | Progressive resistance training. 15–20 repeti- tions in two sets, subjects performed repetitions until momentary failure occurred, 40–50 min per session | SF-36 |
|  |  | 24 | 57.10 ± 16.20 | 28±20 | Control arm | UC/SE | Sham exercise |  |
| Koh 2010[50] | 3 | 15 | 52.3±10.9 | 31±2 | Intervention arm | AE | 15–45 min IDC with Borg’s of RPE scale 12–13 | SF-36 |
|  |  | 16 | 51.3±14.4 |  | Control arm | UC/SE | UC |  |
| Arazi 2021[51] | 3 | 26 | 45.76±2.11 | / | Intervention arm | RMT | Breathing exercise 10 times every hour after wake up with IS | / |
|  |  | 26 | 48.12±3.01 | / | Control arm | UC/SE | UC |  |
| Medeiros 2021[52] | 14 | 12 | 45.50 (39.02–51.98) 95%区间 | 76±44.71 | Intervention arm | RMT | Three sets of 30 breaths with diaphragmatic breathing pattern and 1min rest interval between sets with a load of 50% inspiratory muscle strength | SF-36 |
|  |  | 12 | 47.33 (39.59–55.07) | 87.92±71.76 | Control arm | UC/SE | Sham exercise |  |
| Feldkötter 2021[53] | 3 | 23 | 14.0±3.44 | / | Intervention arm | AE | 50–70 min AE with 70–80% of the patient-specific maximum workload on a bicycle ergometer | / |
|  |  | 22 | 15.1±2.45 | / | Control arm | UC/SE | UC |  |
| Kim 2021[54] | 3 | 18 | 57.61±13.69 | 28.56±32.64 | Intervention arm | AE | 12–15 for 20–50 minutes cycle ergometer exercise on the rate of perceived exertion from the Borg scale | SF-36 |
|  |  | 21 | 56.76±12.32 | 68.88±47.88 | Control arm | UC/SE | UC |  |
| Marini 2021[55] | 3 | 10 | 37.3±9.2 | / | Intervention arm | EMS | Warming up for five minutes at a frequency of 5 Hz, 250 μs, muscle stimulation for 30 min, with frequency of 100 Hz, 400 μs, and relaxation for five minutes at a frequency of 5 Hz, 250 μs | SF-36 |
|  |  | 11 | 45.8±10.8 |  | Control arm | UC/SE | UC |  |
| Myers 2021[56] | 3 | 13 | 66.3±7.6 | 51±46.8 | Intervention arm | AE+RE | Combination of continuous aerobic activities and resistance exercise for a minimum of 45 min per day at 70–80% of HR reserve and 12–14 on the Borg perceived exertion scale | SF-36 |
|  |  | 15 | 66.2±6.7 | 48.6±46.8 | Control arm | UC/SE | UC |  |
| Otobe 2021[57] | 1 | 23 | 78.4±6.4 | / | Intervention arm | AE+RE | 10 min stretching, 20 min RE, 5 min balance exercises, 20 min AE on a cycling ergometer, and 5 min cool down with perceived exertion of 11-13 on the 20-point Borg scale | / |
|  |  | 21 | 78.1±7.4 | / | Control arm | UC/SE | UC |  |
| Borja 2021[58] | 3 | 30 | 67.2±13.3 | / | Intervention arm | AE+RE | 5 min warm-up, strengthening section with different level resistance elastic bands, and 30 min aerobic resistance training with the use of a cycloergometerbased on Borg’s perceived exertion scale | SF-36 |
|  |  | 27 | 67.2±15.9 | / | Control arm | UC/SE | UC |  |

*Abbreviations: SF-36*, Short Form 36; *AE*, Aerobic exercise; *RE*, Resistance exercise; *RT*, Resistance training; *ROM,* Rang of motion; *IDC*, Intradialytic cycling; *UC/SE*, Usual care/Sham exercise; *RPE*, rate of perceived exertion; *EMS*, Electrical muscle stimulation; *W_peak_*, peak workload; *PRT*, progressive resistance training; *1RM*, one repetition maximum; *PMT*, Peripheral muscle training; *RMT*, Respiratory muscle training; *IDE*, Intradialytic exercise; *KDQOL-SF*, Kidney Disease Quality of Life Short Form; *WHOQOL-BREF*, World Health Organization Quality of Life Brief; *WE*, Walking exercise; *RPM*, rounds per minute; *RPMs*, revolutions per minutes; *STE*, Stretching exercise; *RPE*, rate of perceived exertion.

**Table S2** Node-splitting approach for inconsistency assessment of all comparisons

| Side | Direct | | Indirect | | Difference | | P>\|z\| | tau |
| --- | --- | --- | --- | --- | --- | --- | --- | --- |
|  | Coef. | std. Err. | Coef . | std. Err. | Coef. | std. Err. |  |  |
| AB | 13.80164 | 11.95687 | 42.47368 | 35.32875 | -28.672 | 37.42071 | 0.444 | 10.7729 |
| AC* | 45.28091 | 22.23802 | 16.58955 | 29.99405 | 28.69136 | 37.43282 | 0.443 | 10.7733 |
| AF* | 55.68718 | 8.35532 | 0.008793 | 3725.826 | 55.67839 | 3725.836 | 0.988 | 8.93028 |
| AI* | 30.1 | 38.06583 | -29.2534 | 79.72523 | 59.3534 | 94.70132 | 0.531 | 8.36828 |
| AJ* | 58 | 53.52204 | 12.04576 | 89.37645 | 15.95424 | 105.5164 | 0.88 | 9.64879 |
| ВC | -4.499694 | 40.07054 | 24.39965 | 20.71553 | -28.8994 | 45.10853 | 0.522 | 10.0031 |
| BG | -41.89998 | 20.02109 | -29.5724 | 38.24124 | -12.3276 | 43.16493 | 0.775 | 10.8571 |
| CG | -51 | 31.74124 | -63.3279 | 29.25694 | 12.32794 | 43.16798 | 0.775 | 10.8569 |
| CJ* | -14 | 47.85207 | 1.954245 | 98.55209 | -15.9542 | 105.5164 | 0.88 | 9.64879 |
| EF* | -3.48E-08 | 39.1651 | 111.3761 | 7440.713 | -111.376 | 7440.816 | 0.988 | 8.93035 |
| HI* | -34.5 | 37.12857 | 24.8534 | 81.04036 | -59.3534 | 94.70131 | 0.531 | 8.36829 |

**Table S3** Risk of bias of randomized controlled trials

| **Study** | **Randomization sequence generation** | **Allocation concealment** | **Blinding of participants and personnel** | **Blinding of outcome assessment** | **Incomplete outcome data** | **Selective reporting** | **Other sources of bias** |
| --- | --- | --- | --- | --- | --- | --- | --- |
| Depaul 2002 | Low risk | Low risk | Unclear risk | Low risk | High risk | Low risk | Low risk |
| Parsons 2004 | Unclear risk | Unclear risk | Unclear risk | Unclear risk | Low risk | Low risk | Low risk |
| Vilsteren 2005 | Unclear risk | Unclear risk | Unclear risk | Unclear risk | Low risk | Low risk | Low risk |
| Cheema 2007 | Low risk | Low risk | Unclear risk | Low risk | Low risk | Low risk | Low risk |
| Matsumoto 2007 | Unclear risk | Unclear risk | Unclear risk | Unclear risk | Low risk | Low risk | Low risk |
| Ouzouni 2009 | Unclear risk | Unclear risk | Unclear risk | Unclear risk | Low risk | Low risk | Low risk |
| Orti 2009 | Low risk | Low risk | Low risk | Low risk | Low risk | Low risk | Low risk |
| Afshar 2010 | Unclear risk | Unclear risk | Unclear risk | Unclear risk | Low risk | Low risk | Low risk |
| Reboredo 2010 | Unclear risk | Unclear risk | Unclear risk | Unclear risk | Low risk | Low risk | Low risk |
| Dobsak 2011 | Unclear risk | Unclear risk | Unclear risk | Low risk | Low risk | Low risk | Low risk |
| Makhlough 2012 | Low risk | Low risk | Low risk | Unclear risk | Low risk | Low risk | Low risk |
| Song 2012 | Unclear risk | Unclear risk | Unclear risk | Unclear risk | Low risk | Low risk | Low risk |
| Delima 2013 | Low risk | Low risk | Unclear risk | Unclear risk | Low risk | Low risk | Low risk |
| Mohseni 2013 | Low risk | Low risk | High risk | High risk | Low risk | Low risk | Low risk |
| Pellizzaro 2012 | Unclear risk | Unclear risk | Unclear risk | Unclear risk | Low risk | Low risk | Low risk |
| Wu 2014 | Low risk | Low risk | Unclear risk | Unclear risk | Low risk | Low risk | Low risk |
| Liao 2016 | Unclear risk | Unclear risk | Unclear risk | High risk | Low risk | Low risk | Low risk |
| Roxo 2016 | Low risk | Low risk | Unclear risk | Unclear risk | Low risk | Low risk | Low risk |
| Frih 2017 | Low risk | Unclear risk | Low risk | Unclear risk | Low risk | Low risk | Low risk |
| Manfredini 2016 | Unclear risk | Unclear risk | High risk | High risk | High risk | Low risk | Low risk |
| Campos 2018 | Low risk | Low risk | High risk | High risk | Low risk | Low risk | Low risk |
| Mcgregor 2018 | Unclear risk | Unclear risk | Low risk | Low risk | Low risk | Low risk | Low risk |
| Suzuki 2018 | Low risk | Low risk | High risk | Low risk | Low risk | Low risk | Low risk |
| Dong 2019 | Low risk | Low risk | Unclear risk | Unclear risk | Low risk | Low risk | Low risk |
| Fernandes 2019 | Low risk | Low risk | Unclear risk | Unclear risk | Low risk | Low risk | Low risk |
| Hatef 2020 | Low risk | Low risk | Unclear risk | Unclear risk | Low risk | Low risk | Low risk |
| Huang 2020 | Unclear risk | Unclear risk | High risk | Low risk | High risk | Low risk | Low risk |
| Valle 2020 | Low risk | Low risk | Unclear risk | Unclear risk | Low risk | Low risk | Low risk |
| Yeh 2020 | Low risk | Low risk | Unclear risk | Unclear risk | Low risk | Low risk | Low risk |
| Zhang 2020 | Low risk | Low risk | Unclear risk | Low risk | Low risk | Low risk | Low risk |
| Fathi 2021 | Low risk | Low risk | Unclear risk | Unclear risk | Low risk | Low risk | Low risk |
| Greenwood 2021 | Low risk | Low risk | High risk | Low risk | High risk | Low risk | Low risk |
| Lin 2021 | Low risk | Low risk | Unclear risk | Low risk | Low risk | Low risk | Low risk |
| March 2022 | Unclear risk | Unclear risk | High risk | Low risk | Low risk | Low risk | Low risk |
| Rochmawati 2021 | Unclear risk | Unclear risk | Unclear risk | Unclear risk | Low risk | Low risk | Low risk |
| Assawasaksakul 2021 | Low risk | Low risk | Unclear risk | Unclear risk | Low risk | Low risk | Low risk |
| Castaneda 2004 | Unclear risk | Unclear risk | Unclear risk | Low risk | Low risk | Low risk | Low risk |
| Jamshidpour2019 | Low risk | Unclear risk | High risk | Low risk | Low risk | Low risk | Low risk |
| Painter 2002 | High risk | High risk | High risk | Low risk | Low risk | Low risk | Low risk |
| Soliman 2015 | High risk | High risk | Unclear risk | Unclear risk | Low risk | Low risk | Low risk |
| Abreu 2017 | Unclear risk | Unclear risk | Unclear risk | Unclear risk | Low risk | Low risk | Low risk |
| Hristea 2016 | Unclear risk | Unclear risk | High risk | Unclear risk | Low risk | Low risk | Low risk |
| Koufaki 2002 | Low risk | High risk | Unclear risk | Unclear risk | Low risk | Low risk | Low risk |
| Kouidi 2009 | Unclear risk | Unclear risk | Unclear risk | Unclear risk | Low risk | Low risk | Low risk |
| Marchesan 2014 | Low risk | High risk | Unclear risk | Unclear risk | Low risk | Low risk | Low risk |
| Marchesan 2016 | Unclear risk | Unclear risk | Unclear risk | Unclear risk | Low risk | Low risk | Low risk |
| Schardong 2017 | Low risk | Low risk | Unclear risk | Unclear risk | Low risk | Low risk | Low risk |
| Poorsaadet 2018 | Unclear risk | Unclear risk | Unclear risk | Unclear risk | Low risk | Low risk | Low risk |
| Rosa 2018 | Unclear risk | Unclear risk | High risk | Unclear risk | Low risk | Low risk | Low risk |
| Koh 2010 | Low risk | Low risk | High risk | Low risk | Low risk | Low risk | Low risk |
| Arazi 2021 | Low risk | Low risk | High risk | Low risk | Low risk | Low risk | Low risk |
| Medeiros 2021 | Low risk | Low risk | Low risk | Low risk | Low risk | Low risk | Low risk |
| Feldkötter 2021 | Low risk | Low risk | High risk | Unclear risk | Low risk | Low risk | Low risk |
| Kim 2021 | Unclear risk | Unclear risk | High risk | Unclear risk | Low risk | Low risk | Low risk |
| Marini 2021 | Unclear risk | Unclear risk | Unclear risk | Unclear risk | Low risk | Low risk | Low risk |
| Myers 2021 | Unclear risk | Unclear risk | Unclear risk | Low risk | Low risk | Low risk | Low risk |
| Otobe 2021 | Low risk | Low risk | High risk | Low risk | Low risk | Low risk | Low risk |
| Borja 2021 | Low risk | Low risk | Low risk | Low risk | High risk | Low risk | Low risk |
| Kappa | 0.969 | 0.740 | 0.980 | 0.913 | 0.818 | 1.000 | 1.000 |



**Figure S1** - Quality assessment of the included studies. (A) Summary of bias risk. (B) Risk of bias for each included study. “+” represents low risk of bias; “–” represents high risk of bias; and “?” represents unclear risk of bias

**A**

**B**

**Figure S2 – Loop-specific approach for inconsistency assessment of all comparisons. (A) six-minute walk test, (B) quality of life, (C) c-reactive protein, (D) VO2max, (E) hemoglobin, (F) Kt/V, (G) systolic blood pressure, (H) diastolic blood pressure comparing different modalities of exercise**

**A**


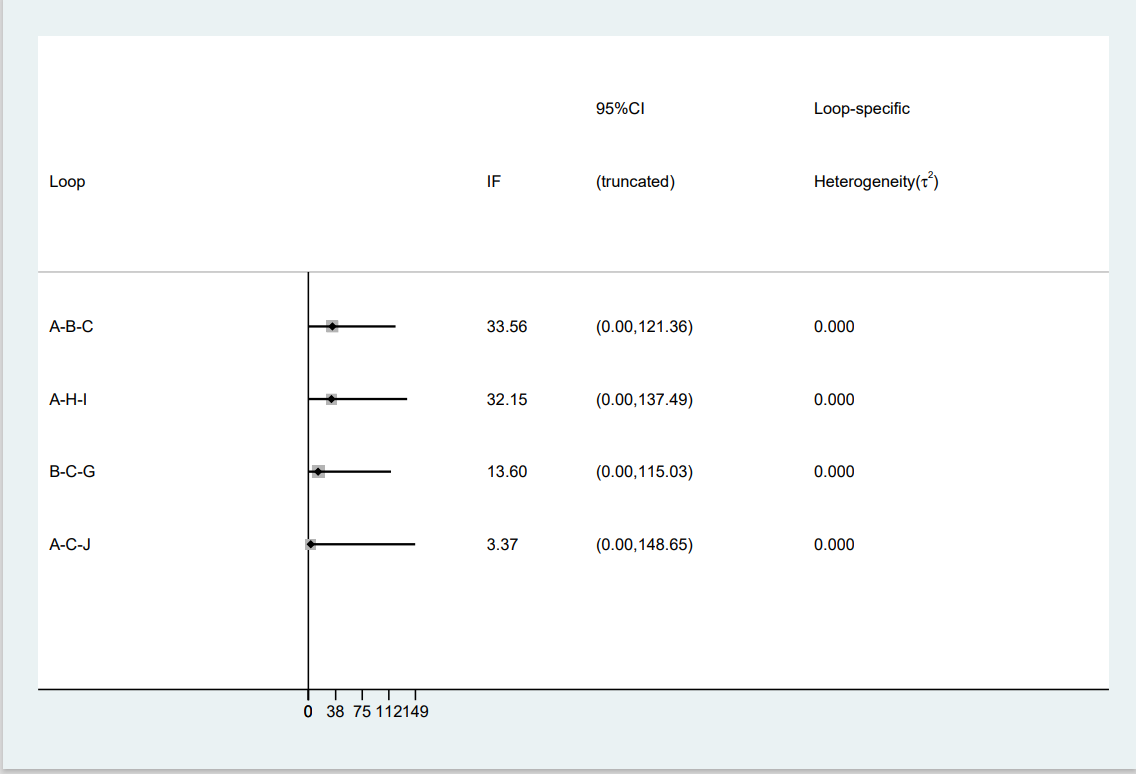


**B**


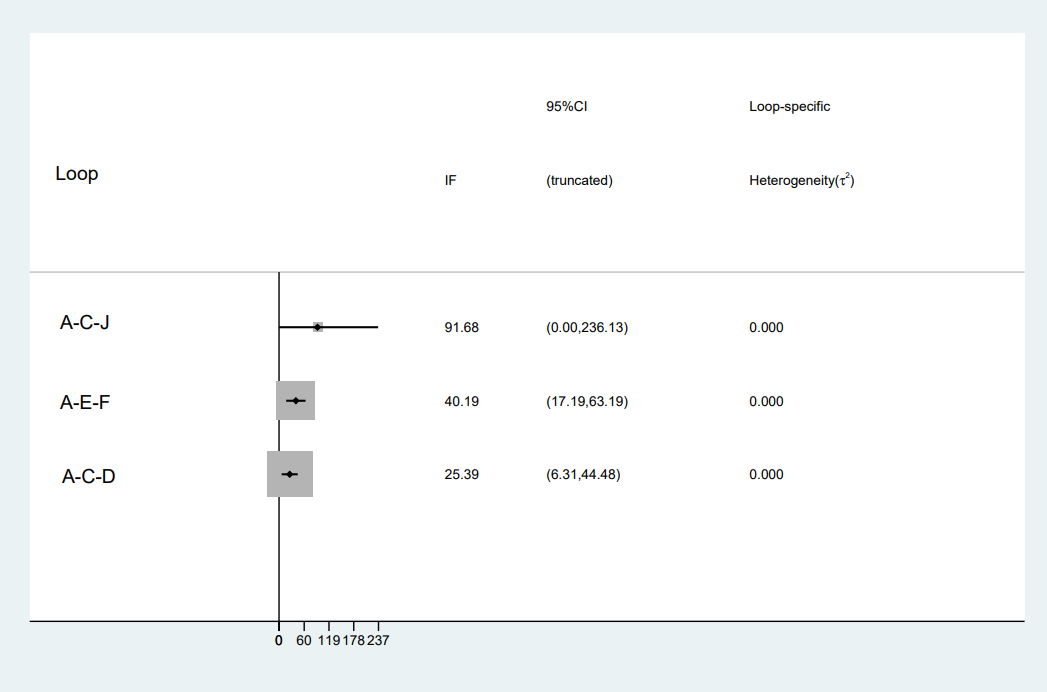


**C**


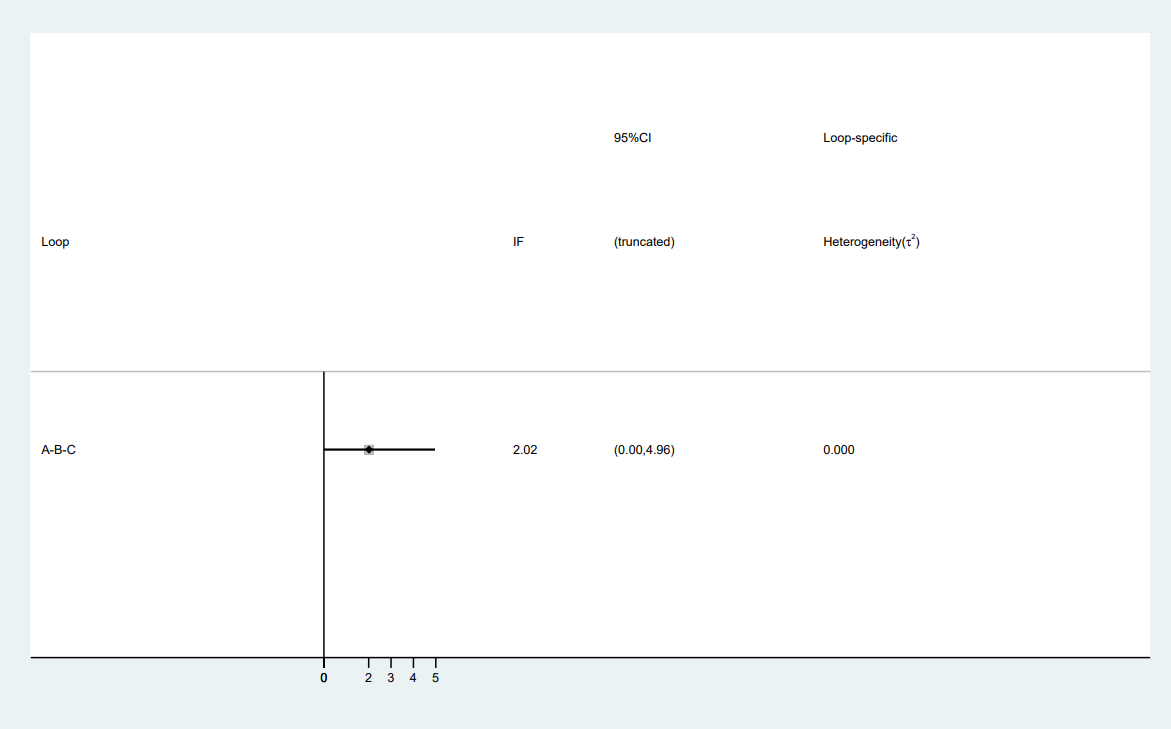


**D**


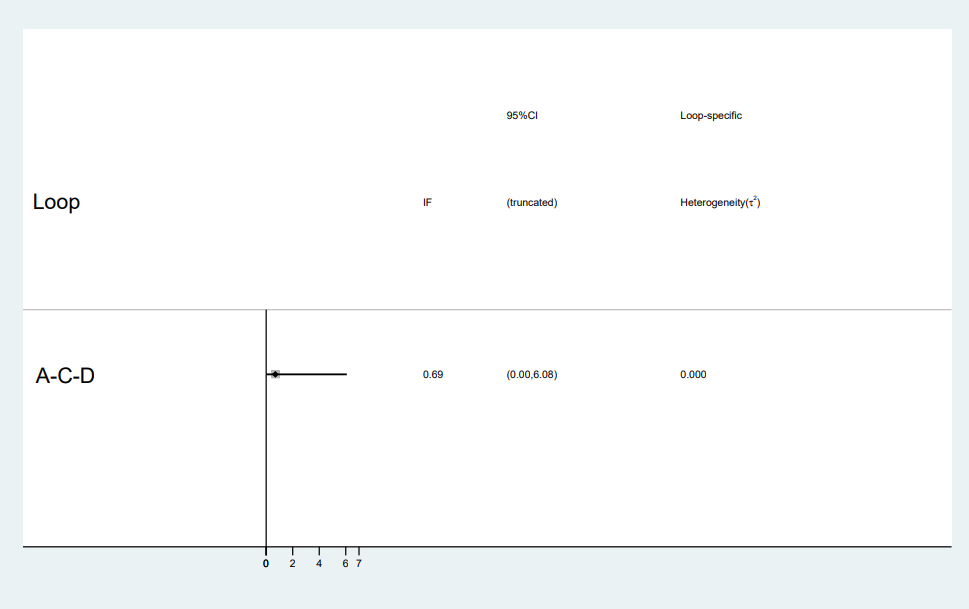


**E**


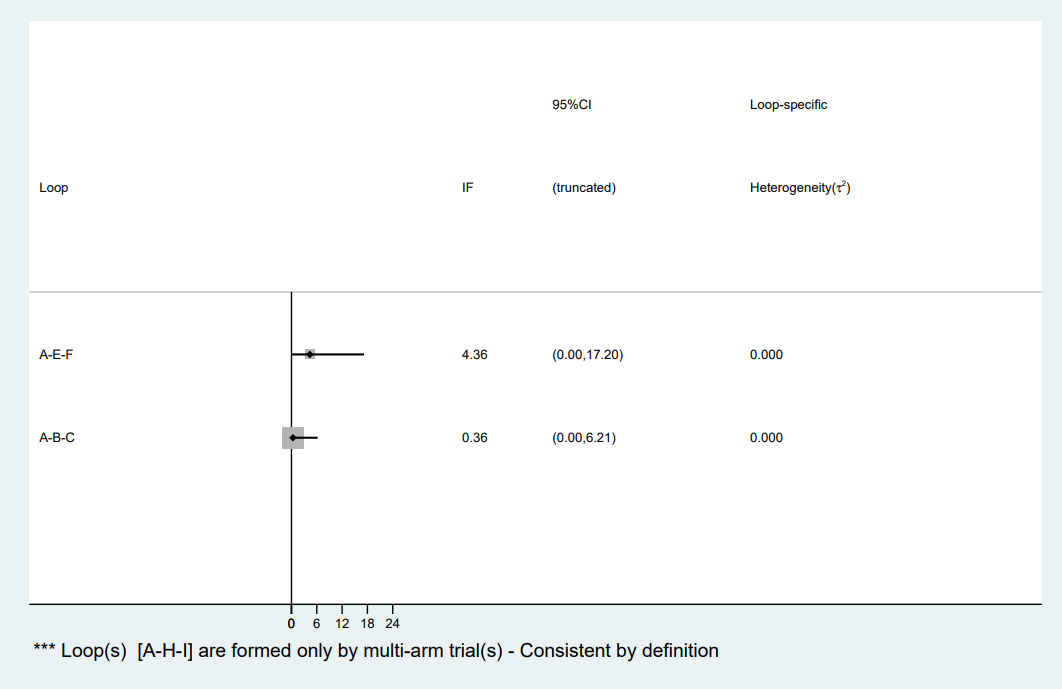


**F**


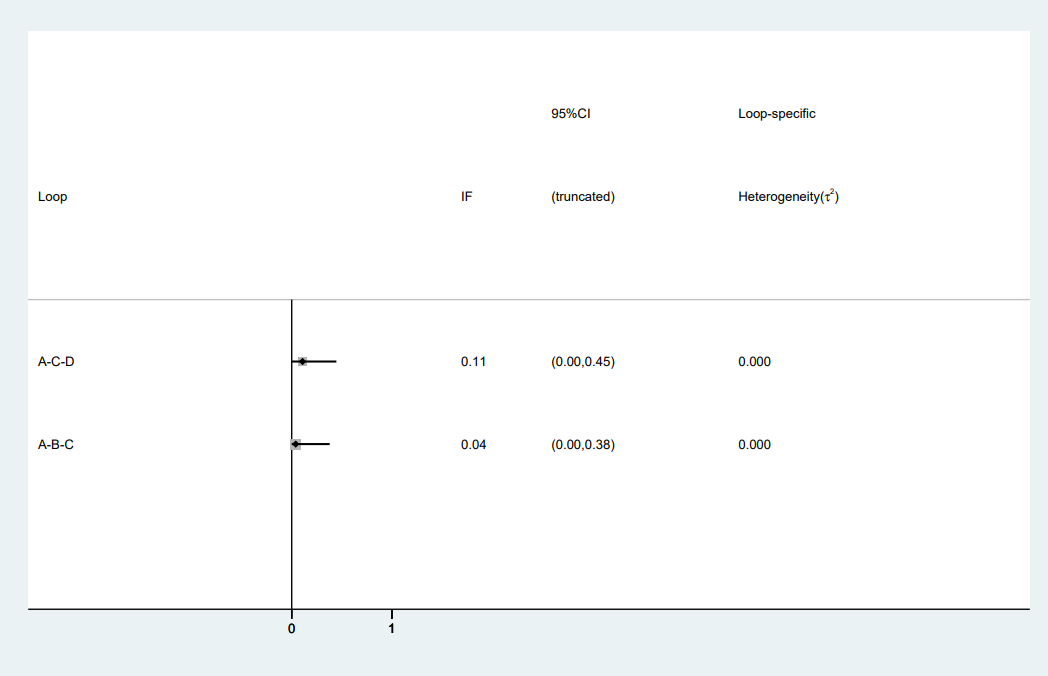


**G**


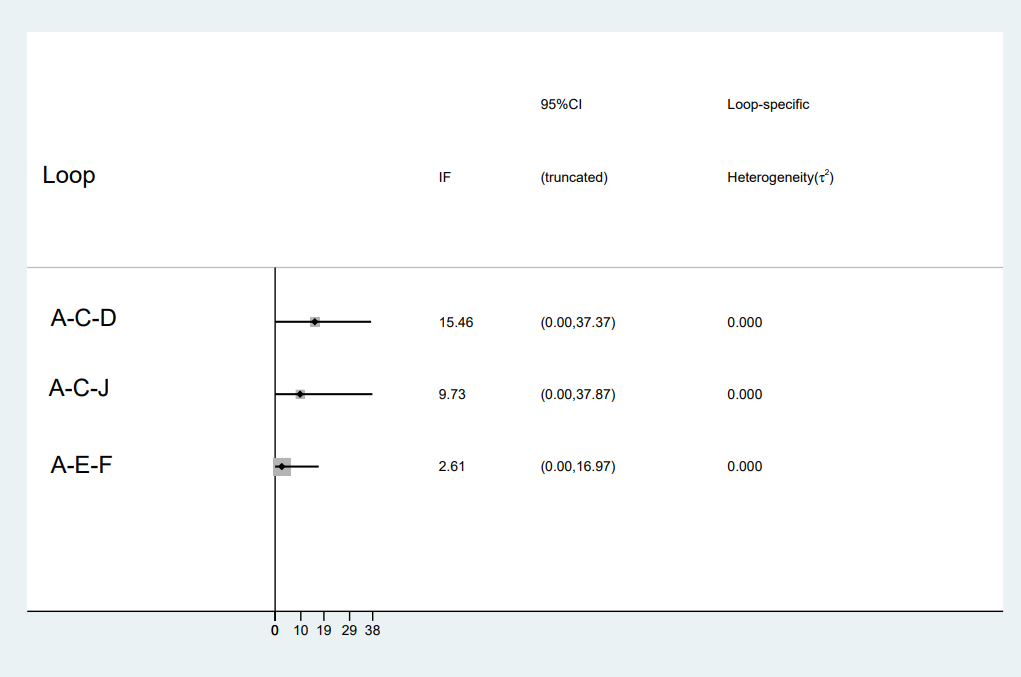


**H**


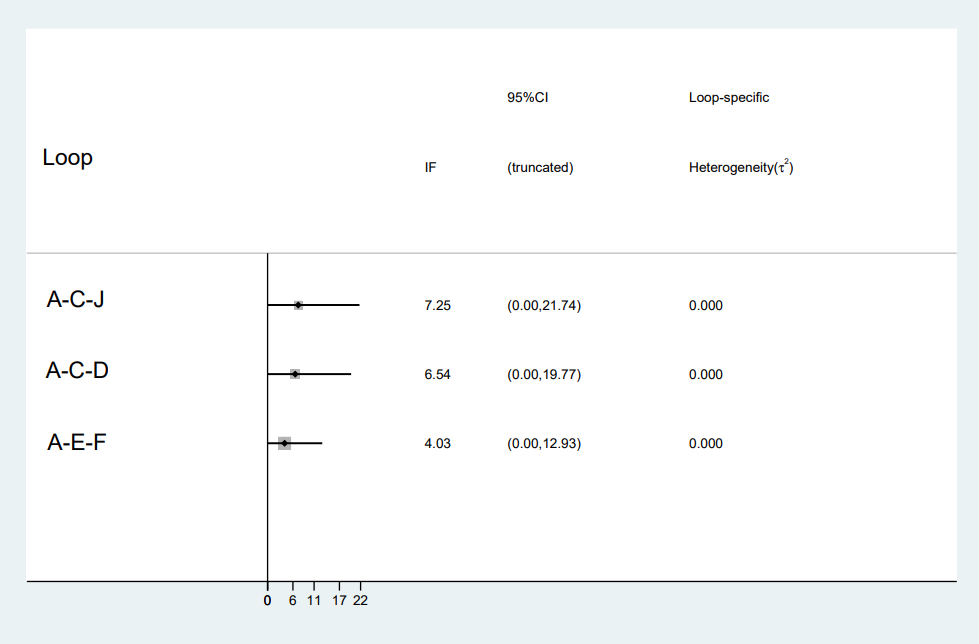


**Figure S3 –** Network of physical exercises for IL-6


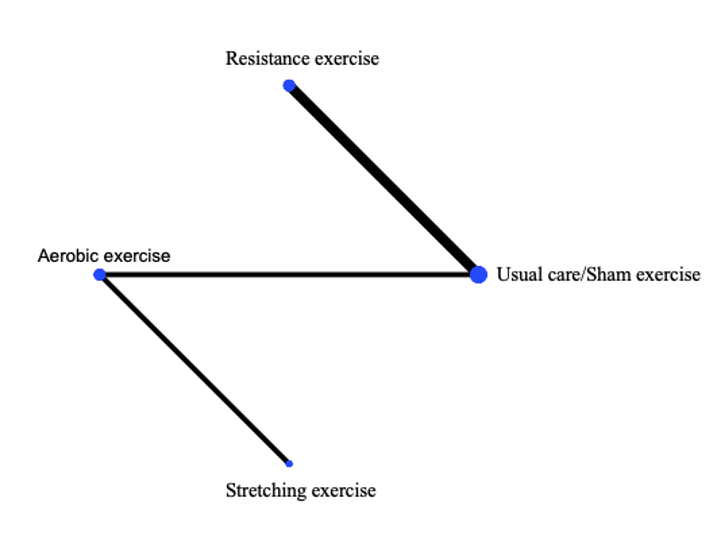


**Figure S4 –** Forest plots of network meta-analysis for IL-6


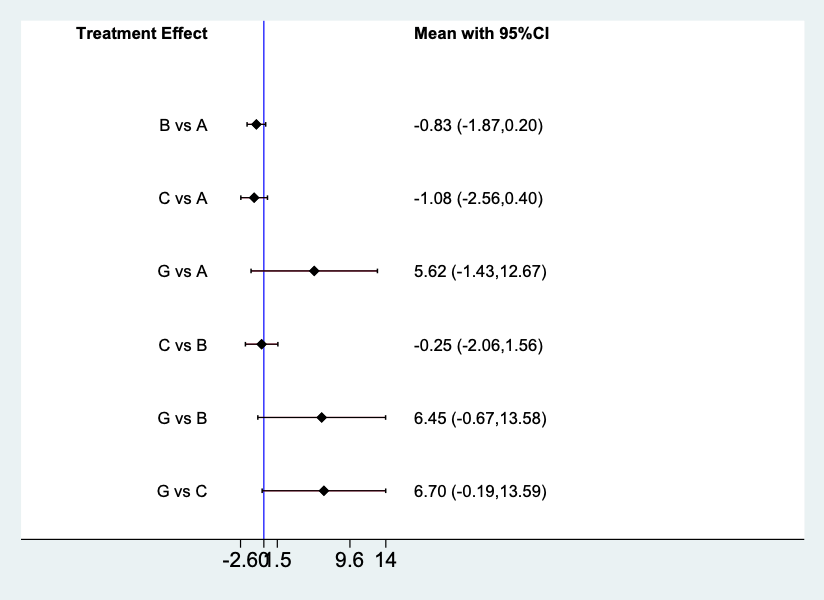


**Figure S5 –** The surface under the cumulative ranking curve (SUCRA) for physical exercise interventions for IL-6


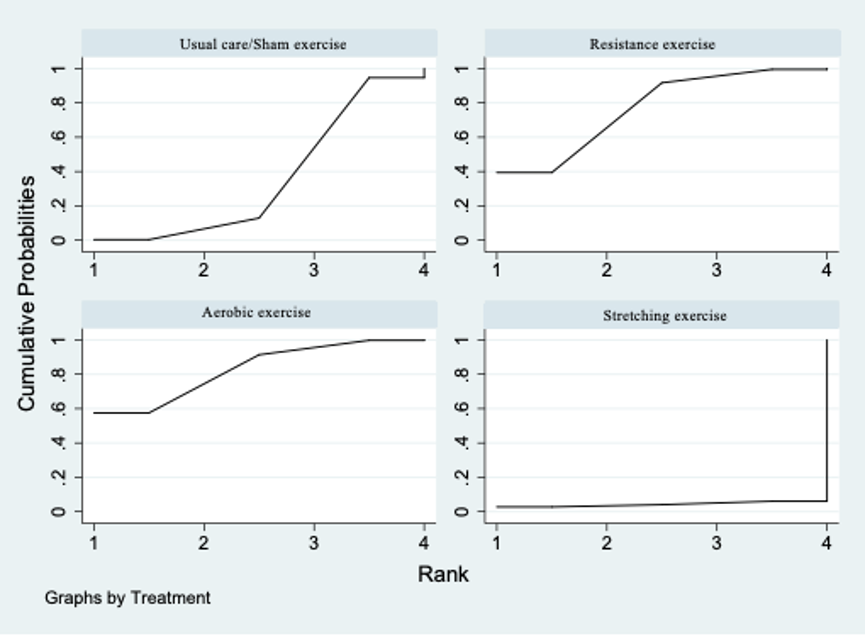


**Figure S6** – Funnel plots of the study treatments for IL-6


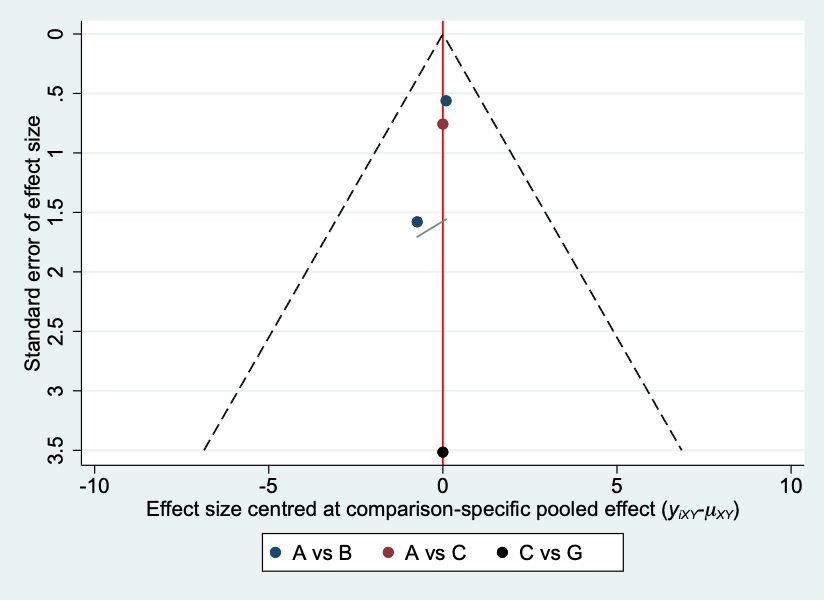


Reference

[1] V. DePaul, J. Moreland, T. Eager, and C.M. Clase, The effectiveness of aerobic and muscle strength training in patients receiving hemodialysis and EPO: a randomized controlled trial. American journal of kidney diseases : the official journal of the National Kidney Foundation 40 (2002) 1219-29.

[2] T.L. Parsons, E.B. Toffelmire, and C.E. King-VanVlack, The effect of an exercise program during hemodialysis on dialysis efficacy, blood pressure and quality of life in end-stage renal disease (ESRD) patients. Clinical nephrology 61 (2004) 261-74.

[3] M.C. van Vilsteren, M.H. de Greef, and R.M. Huisman, The effects of a low-to-moderate intensity pre-conditioning exercise programme linked with exercise counselling for sedentary haemodialysis patients in The Netherlands: results of a randomized clinical trial. Nephrology, dialysis, transplantation : official publication of the European Dialysis and Transplant Association - European Renal Association 20 (2005) 141-6.

[4] B. Cheema, H. Abas, B. Smith, A. O'Sullivan, M. Chan, A. Patwardhan, J. Kelly, A. Gillin, G. Pang, B. Lloyd, and M.F. Singh, Progressive exercise for anabolism in kidney disease (PEAK): a randomized, controlled trial of resistance training during hemodialysis. Journal of the American Society of Nephrology : JASN 18 (2007) 1594-601.

[5] Y. Matsumoto, A. Furuta, S. Furuta, M. Miyajima, T. Sugino, K. Nagata, and S. Sawada, The impact of pre-dialytic endurance training on nutritional status and quality of life in stable hemodialysis patients (Sawada study). Renal failure 29 (2007) 587-93.

[6] S. Ouzouni, E. Kouidi, A. Sioulis, D. Grekas, and A. Deligiannis, Effects of intradialytic exercise training on health-related quality of life indices in haemodialysis patients. Clinical rehabilitation 23 (2009) 53-63.

[7] E. Segura-Ortí, E. Kouidi, and J.F. Lisón, Effect of resistance exercise during hemodialysis on physical function and quality of life: randomized controlled trial. Clinical nephrology 71 (2009) 527-37.

[8] R. Afshar, L. Shegarfy, N. Shavandi, and S. Sanavi, Effects of aerobic exercise and resistance training on lipid profiles and inflammation status in patients on maintenance hemodialysis. Indian journal of nephrology 20 (2010) 185-9.

[9] M. Reboredo Mde, V. Pinheiro Bdo, J.A. Neder, M.P. Ávila, E.R.M.L. Araujo, A.F. de Mendonça, M.V. de Mello, A.C. Bainha, J. Dondici Filho, and R.B. de Paula, Effects of aerobic training during hemodialysis on heart rate variability and left ventricular function in end-stage renal disease patients. Jornal brasileiro de nefrologia : 'orgao oficial de Sociedades Brasileira e Latino-Americana de Nefrologia 32 (2010) 367-73.

[10] P. Dobsak, P. Homolka, J. Svojanovsky, A. Reichertova, M. Soucek, M. Novakova, L. Dusek, J. Vasku, J.C. Eicher, and J. Siegelova, Intra-dialytic electrostimulation of leg extensors may improve exercise tolerance and quality of life in hemodialyzed patients. Artificial organs 36 (2012) 71-8.

[11] A. Makhlough, E. Ilali, R. Mohseni, and S. Shahmohammadi, Effect of intradialytic aerobic exercise on serum electrolytes levels in hemodialysis patients. Iranian journal of kidney diseases 6 (2012) 119-23.

[12] W.J. Song, and K.Y. Sohng, Effects of progressive resistance training on body composition, physical fitness and quality of life of patients on hemodialysis. Journal of Korean Academy of Nursing 42 (2012) 947-56.

[13] M.C. de Lima, L. Cicotoste Cde, S. Cardoso Kda, L.A. Forgiarini, Jr., M.B. Monteiro, and A.S. Dias, Effect of exercise performed during hemodialysis: strength versus aerobic. Renal failure 35 (2013) 697-704.

[14] R. Mohseni, A. Emami Zeydi, E. Ilali, M. Adib-Hajbaghery, and A. Makhlough, The effect of intradialytic aerobic exercise on dialysis efficacy in hemodialysis patients: a randomized controlled trial. Oman medical journal 28 (2013) 345-9.

[15] C.O. Pellizzaro, F.S. Thomé, and F.V. Veronese, Effect of peripheral and respiratory muscle training on the functional capacity of hemodialysis patients. Renal failure 35 (2013) 189-97.

[16] Y. Wu, Q. He, X. Yin, Q. He, S. Cao, and G. Ying, Effect of individualized exercise during maintenance haemodialysis on exercise capacity and health-related quality of life in patients with uraemia. The Journal of international medical research 42 (2014) 718-27.

[17] M.T. Liao, W.C. Liu, F.H. Lin, C.F. Huang, S.Y. Chen, C.C. Liu, S.H. Lin, K.C. Lu, and C.C. Wu, Intradialytic aerobic cycling exercise alleviates inflammation and improves endothelial progenitor cell count and bone density in hemodialysis patients. Medicine 95 (2016) e4134.

[18] R.S. Roxo, V.B. Xavier, L.A. Miorin, A.O. Magalhães, Y.A. Sens, and V.L. Alves, Impact of neuromuscular electrical stimulation on functional capacity of patients with chronic kidney disease on hemodialysis. Jornal brasileiro de nefrologia : 'orgao oficial de Sociedades Brasileira e Latino-Americana de Nefrologia 38 (2016) 344-350.

[19] B. Frih, H. Jaafar, W. Mkacher, Z. Ben Salah, M. Hammami, and A. Frih, The Effect of Interdialytic Combined Resistance and Aerobic Exercise Training on Health Related Outcomes in Chronic Hemodialysis Patients: The Tunisian Randomized Controlled Study. Frontiers in physiology 8 (2017) 288.

[20] F. Manfredini, F. Mallamaci, G. D'Arrigo, R. Baggetta, D. Bolignano, C. Torino, N. Lamberti, S. Bertoli, D. Ciurlino, L. Rocca-Rey, A. Barillà, Y. Battaglia, R.M. Rapanà, A. Zuccalà, G. Bonanno, P. Fatuzzo, F. Rapisarda, S. Rastelli, F. Fabrizi, P. Messa, L. De Paola, L. Lombardi, A. Cupisti, G. Fuiano, G. Lucisano, C. Summaria, M. Felisatti, E. Pozzato, A.M. Malagoni, P. Castellino, F. Aucella, S. Abd ElHafeez, P.F. Provenzano, G. Tripepi, L. Catizone, and C. Zoccali, Exercise in Patients on Dialysis: A Multicenter, Randomized Clinical Trial. Journal of the American Society of Nephrology : JASN 28 (2017) 1259-1268.

[21] N.G. Campos, D.F. Marizeiro, A.C.L. Florêncio, C. Silva Í, G.C. Meneses, G.F. Bezerra, A.M.C. Martins, and A.B. Libório, Effects of respiratory muscle training on endothelium and oxidative stress biomarkers in hemodialysis patients: A randomized clinical trial. Respiratory medicine 134 (2018) 103-109.

[22] G. McGregor, S. Ennis, R. Powell, T. Hamborg, N.T. Raymond, W. Owen, N. Aldridge, G. Evans, J. Goodby, S. Hewins, P. Banerjee, N.S. Krishnan, S.M.S. Ting, and D. Zehnder, Feasibility and effects of intra-dialytic low-frequency electrical muscle stimulation and cycle training: A pilot randomized controlled trial. PloS one 13 (2018) e0200354.

[23] T. Suzuki, M. Ikeda, M. Minami, Y. Matayoshi, M. Nakao, T. Nakamura, and M. Abo, Beneficial Effect of Intradialytic Electrical Muscle Stimulation in Hemodialysis Patients: A Randomized Controlled Trial. Artificial organs 42 (2018) 899-910.

[24] Z.J. Dong, H.L. Zhang, and L.X. Yin, Effects of intradialytic resistance exercise on systemic inflammation in maintenance hemodialysis patients with sarcopenia: a randomized controlled trial. International urology and nephrology 51 (2019) 1415-1424.

[25] A.O. Fernandes, Y. Sens, V.B. Xavier, L.A. Miorin, and V. Alves, Functional and Respiratory Capacity of Patients with Chronic Kidney Disease Undergoing Cycle Ergometer Training during Hemodialysis Sessions: A Randomized Clinical Trial. International journal of nephrology 2019 (2019) 7857824.

[26] M. Hatef, N. Mousavinasab, R. Esmaeili, M. Kamali, Z. Madani, F. Spahbodi, and V. Shafipour, The Effects of Exercise Training on Physical Performance and Self-efficacy in Hemodialysis Patients: A Randomized Controlled Clinical Trial. Iranian journal of nursing and midwifery research 25 (2020) 520-526.

[27] M. Huang, A. Lv, J. Wang, B. Zhang, N. Xu, Z. Zhai, J. Gao, Y. Wang, T. Li, and C. Ni, The effect of intradialytic combined exercise on hemodialysis efficiency in end-stage renal disease patients: a randomized-controlled trial. International urology and nephrology 52 (2020) 969-976.

[28] F. Martins do Valle, B. Valle Pinheiro, A.A. Almeida Barros, W. Ferreira Mendonça, A.C. de Oliveira, G. de Oliveira Werneck, R.B. de Paula, and M. Moura Reboredo, Effects of intradialytic resistance training on physical activity in daily life, muscle strength, physical capacity and quality of life in hemodialysis patients: a randomized clinical trial. Disability and rehabilitation 42 (2020) 3638-3644.

[29] M.L. Yeh, M.H. Wang, C.C. Hsu, and Y.M. Liu, Twelve-week intradialytic cycling exercise improves physical functional performance with gain in muscle strength and endurance: a randomized controlled trial. Clinical rehabilitation 34 (2020) 916-926.

[30] F. Zhang, L. Huang, W. Wang, Q. Shen, and H. Zhang, Effect of intradialytic progressive resistance exercise on physical fitness and quality of life in maintenance haemodialysis patients. Nursing open 7 (2020) 1945-1953.

[31] M. Fathi, and K. Hejazi, The effect of six months aerobic exercise during dialysis on liver enzymes, cystatin C and quality of life of hemodialysis patients. The Journal of sports medicine and physical fitness 61 (2021) 1515-1522.

[32] S.A. Greenwood, P. Koufaki, J.H. Macdonald, C. Bulley, S. Bhandari, J.O. Burton, I. Dasgupta, K. Farrington, I. Ford, P.A. Kalra, M. Kumwenda, I.C. Macdougall, C.M. Messow, S. Mitra, C. Reid, A.C. Smith, M.W. Taal, P.C. Thomson, D.C. Wheeler, C. White, M. Yaqoob, and T.H. Mercer, Exercise programme to improve quality of life for patients with end-stage kidney disease receiving haemodialysis: the PEDAL RCT. Health technology assessment (Winchester, England) 25 (2021) 1-52.

[33] C.H. Lin, Y.J. Hsu, P.H. Hsu, Y.L. Lee, C.H. Lin, M.S. Lee, and S.L. Chiang, Effects of Intradialytic Exercise on Dialytic Parameters, Health-Related Quality of Life, and Depression Status in Hemodialysis Patients: A Randomized Controlled Trial. International journal of environmental research and public health 18 (2021).

[34] D.S. March, K.B. Lai, T. Neal, M.P.M. Graham-Brown, P.J. Highton, D.R. Churchward, H.M.L. Young, M. Dungey, D.J. Stensel, A.C. Smith, N.C. Bishop, C.C. Szeto, and J.O. Burton, Circulating endotoxin and inflammation: associations with fitness, physical activity and the effect of a 6-month programme of cycling exercise during haemodialysis. Nephrology, dialysis, transplantation : official publication of the European Dialysis and Transplant Association - European Renal Association 37 (2022) 366-374.

[35] E. Rochmawati, E.K. Utomo, and S.N.N. Makiyah, Improving dialysis adequacy and quality of life in patients undergoing hemodialysis with twice a week range of motion exercise. Therapeutic apheresis and dialysis : official peer-reviewed journal of the International Society for Apheresis, the Japanese Society for Apheresis, the Japanese Society for Dialysis Therapy 26 (2022) 140-146.

[36] N. Assawasaksakul, W. Sirichana, W. Joosri, O. Kulaputana, S. Eksakulkla, C. Ketanun, P. Kittiskulnam, M. Chantadisai, K. Takkavatakarn, P. Susantitaphong, K. Praditpornsilpa, S. Eiam-Ong, and K. Tiranathanagul, Effects of intradialytic cycling exercise on daily physical activity, physical fitness, body composition, and clinical parameters in high-volume online hemodiafiltration patients: a pilot randomized-controlled trial. International urology and nephrology 53 (2021) 359-371.

[37] C. Castaneda, P.L. Gordon, R.C. Parker, K.L. Uhlin, R. Roubenoff, and A.S. Levey, Resistance training to reduce the malnutrition-inflammation complex syndrome of chronic kidney disease. American journal of kidney diseases : the official journal of the National Kidney Foundation 43 (2004) 607-16.

[38] B. Jamshidpour, F. Bahrpeyma, and M.R. Khatami, The effect of aerobic and resistance exercise training on the health related quality of life, physical function, and muscle strength among hemodialysis patients with Type 2 diabetes. Journal of bodywork and movement therapies 24 (2020) 98-103.

[39] P. Painter, G. Moore, L. Carlson, S. Paul, J. Myll, W. Phillips, and W. Haskell, Effects of exercise training plus normalization of hematocrit on exercise capacity and health-related quality of life. American journal of kidney diseases : the official journal of the National Kidney Foundation 39 (2002) 257-65.

[40] H.M.M. Soliman, Effect of intradialytic exercise on fatigue, electrolytes level and blood pressure in hemodialysis patients: a randomized controlled trial. Journal of Nursing Education and Practice 5 (2015) 16-28.

[41] C.C. Abreu, L. Cardozo, M.B. Stockler-Pinto, M. Esgalhado, J.E. Barboza, R. Frauches, and D. Mafra, Does resistance exercise performed during dialysis modulate Nrf2 and NF-κB in patients with chronic kidney disease? Life sciences 188 (2017) 192-197.

[42] D. Hristea, T. Deschamps, A. Paris, G. Lefrançois, V. Collet, C. Savoiu, S. Ozenne, S. Coupel, A. Testa, and J. Magnard, Combining intra-dialytic exercise and nutritional supplementation in malnourished older haemodialysis patients: Towards better quality of life and autonomy. Nephrology (Carlton, Vic.) 21 (2016) 785-90.

[43] P. Koufaki, T.H. Mercer, and P.F. Naish, Effects of exercise training on aerobic and functional capacity of end-stage renal disease patients. Clinical physiology and functional imaging 22 (2002) 115-24.

[44] E. Kouidi, V. Karagiannis, D. Grekas, A. Iakovides, G. Kaprinis, A. Tourkantonis, and A. Deligiannis, Depression, heart rate variability, and exercise training in dialysis patients. European journal of cardiovascular prevention and rehabilitation : official journal of the European Society of Cardiology, Working Groups on Epidemiology & Prevention and Cardiac Rehabilitation and Exercise Physiology 17 (2010) 160-7.

[45] M. Marchesan, V.G.d.S. Nunes, and A.J. Rombaldi, Physical training improves physical fitness and the quality of life of patients on hemodialysis. Revista Brasileira de Cineantropometria & Desempenho Humano 16 (2014) 334-344.

[46] M. Marchesan, R.d.R. Krug, J.R.L.d.C. Silva, A.R. Barbosa, and A.J. Rombaldi, Physical exercise modifies the functional capacity of elderly patients on hemodialysis. Fisioterapia em Movimento 29 (2016) 351-359.

[47] J. Schardong, T. Dipp, C.B. Bozzeto, M.G. da Silva, G.L. Baldissera, R.C. Ribeiro, B.P. Valdemarca, A.S. do Pinho, G. Sbruzzi, and R.D.M. Plentz, Effects of Intradialytic Neuromuscular Electrical Stimulation on Strength and Muscle Architecture in Patients With Chronic Kidney Failure: Randomized Clinical Trial. Artificial organs 41 (2017) 1049-1058.

[48] L. Poorsaadet, P. Soltani, K. Ghassami, B. Kohansal, and M. Ahmadlou, The effects of aerobic exercise on cognitive performance and sleep quality haemodialysis patients. Australasian Medical Journal (Online) 11 (2018) 278-285.

[49] C. Rosa, D.Y. Nishimoto, G.D.E. Souza, A.P. Ramirez, C.O. Carletti, C.G.L. Daibem, G.K. Sakkas, and H.L. Monteiro, Effect of continuous progressive resistance training during hemodialysis on body composition, physical function and quality of life in end-stage renal disease patients: a randomized controlled trial. Clinical rehabilitation 32 (2018) 899-908.

[50] K.P. Koh, R.G. Fassett, J.E. Sharman, J.S. Coombes, and A.D. Williams, Effect of intradialytic versus home-based aerobic exercise training on physical function and vascular parameters in hemodialysis patients: a randomized pilot study. American journal of kidney diseases : the official journal of the National Kidney Foundation 55 (2010) 88-99.

[51] T. Arazi, M. Aliasgharpour, S. Mohammadi, N. Mohammadi, and A. Kazemnejad, Effect of a Breathing Exercise on Respiratory Function and 6-Minute Walking Distance in Patients Under Hemodialysis: A Randomized Controlled Trial. The journal of nursing research : JNR 29 (2021) e146.

[52] A.I.C. Medeiros, D.C. Brandão, R.J.P. Souza, H.K.B. Fuzari, C. Barros, J.B.N. Barbosa, J.C. Leite, F.C.B. Cavalcanti, A. Dornelas de Andrade, and P. de Melo Marinho, Effects of daily inspiratory muscle training on respiratory muscle strength and chest wall regional volumes in haemodialysis patients: a randomised clinical trial. Disability and rehabilitation 41 (2019) 3173-3180.

[53] M. Feldkötter, S. Thys, A. Adams, I. Becker, R. Büscher, M. Pohl, R. Schild, L. Pape, C.P. Schmitt, C. Taylan, S. Wygoda, G. Klaus, H. Fehrenbach, C. Montoya, M. Konrad, H. Billing, B. Schaar, and B. Hoppe, Endurance-oriented training program with children and adolescents on maintenance hemodialysis to enhance dialysis efficacy-DiaSport. Pediatric nephrology (Berlin, Germany) 36 (2021) 3923-3932.

[54] S. Kim, H.J. Park, and D.H. Yang, An intradialytic aerobic exercise program ameliorates frailty and improves dialysis adequacy and quality of life among hemodialysis patients: a randomized controlled trial. Kidney research and clinical practice 41 (2022) 462-472.

[55] A.C.B. Marini, R.D. Motobu, P.C.B. Lobo, P.A. Monteiro, and G.D. Pimentel, No effect of intradialytic neuromuscular electrical stimulation on inflammation and quality of life: a randomized and parallel design clinical trial. Scientific reports 11 (2021) 22176.

[56] J. Myers, K. Chan, Y. Chen, Y. Lit, A. Patti, P. Massaband, B.J. Kiratli, M. Tamura, G.M. Chertow, and R. Rabkin, Effect of a Home-Based Exercise Program on Indices of Physical Function and Quality of Life in Elderly Maintenance Hemodialysis Patients. Kidney & blood pressure research 46 (2021) 196-206.

[57] Y. Otobe, M. Yamada, K. Hiraki, S. Onari, Y. Taki, H. Sumi, R. Hachisuka, W. Han, M. Takahashi, M. Suzuki, Y. Kimura, S. Koyama, H. Masuda, Y. Shibagaki, and N. Tominaga, Physical Exercise Improves Cognitive Function in Older Adults with Stage 3-4 Chronic Kidney Disease: A Randomized Controlled Trial. American journal of nephrology 52 (2021) 929-939.

[58] B. Perez-Dominguez, J. Casaña-Granell, R. Garcia-Maset, A. Garcia-Testal, E. Melendez-Oliva, and E. Segura-Orti, Effects of exercise programs on physical function and activity levels in patients undergoing hemodialysis: a randomized controlled trial. European journal of physical and rehabilitation medicine 57 (2021) 994-1001.
